# Network-Based Approach to Identify Potential Targets and Drugs that Promote Neuroprotection and Neurorepair in Acute Ischemic Stroke

**DOI:** 10.1038/srep40137

**Published:** 2017-01-05

**Authors:** Yiwei Wang, Hailong Liu, Yongzhong Lin, Guangming Liu, Hongwei Chu, Pengyao Zhao, Xiaohan Yang, Tiezheng Zheng, Ming Fan, Xuezhong Zhou, Jun Meng, Changkai Sun

**Affiliations:** 1 Department of Biomedical Engineering, Faculty of Electronic Information and Electrical Engineering, Dalian University of Technology, Dalian 116024, China; 2 Liaoning Provincial Key Laboratory of Cerebral Diseases, Institute for Brain Disorders, Dalian Medical University, Dalian 116044, China; 3 Department of Neurology, the Second Affiliated Hospital of Dalian Medical University, Dalian 116027, China; 4 School of Computer and Information Technology and Beijing Key Lab of Traffic Data Analysis and Mining, Beijing Jiaotong University, Beijing 100044, China; 5 Institute of Basic Medical Sciences, Academy of Military Medical Sciences, Beijing 100850, China; 6 College of Electrical Engineering, Zhejiang University, Hangzhou 310027, China; 7 Research Center for the Control Engineering of Translational Precision Medicine, Dalian University of Technology, Dalian 116024, China; 8 State Key Laboratory of Fine Chemicals, Dalian R&D Center for Stem Cell and Tissue Engineering, Dalian University of Technology, Dalian 116024, China

## Abstract

Acute ischemic stroke (AIS) accounts for more than 80% of the approximately 610,000 new stroke cases worldwide every year. Both ischemia and reperfusion can cause death, damage, and functional changes of affected nerve cells, and these alterations can result in high rates of disability and mortality. Therefore, therapies aimed at increasing neuroprotection and neurorepair would make significant contributions to AIS management. However, with regard to AIS therapies, there is currently a large gap between experimental achievements and practical clinical solutions (EC-GAP-AIS). Here, by integrating curated disease-gene associations and interactome network known to be related to AIS, we investigated the molecular network mechanisms of multi-module structures underlying AIS, which might be relevant to the time frame subtypes of AIS. In addition, the EC-GAP-AIS phenomenon was confirmed and elucidated by the shortest path lengths and the inconsistencies in the molecular functionalities and overlapping pathways between AIS-related genes and drug targets. Furthermore, we identified 23 potential targets (e.g. ADORA3, which is involved in the regulation of cellular reprogramming and the extracellular matrix) and 46 candidate drugs (e.g. felbamate, methylphenobarbital and memantine) that may have value for the treatment of AIS.

Acute ischemic stroke (AIS) is a disease that is characterized by neuronal dysfunction and apoptosis induced by the interruption of blood supply resulting from the occlusion or rupture of blood vessels^1^. It is the most common cause of death and a major cause of disability worldwide^2^. Each year, 795,000 people experience a new or recurrent stroke. Approximately 610,000 of these strokes are first attacks, of which 87% are ischemic^3^. 5 years after a stroke, approximately 47% of patients died, and more than one-third of all survivors are left disabled^4^. In the United States, the costs associated with treatment for ischemic stroke are large financial burden, totaling more than $70 billion^5^. The high rates of stroke-associated mortality and disability result from neuronal injury^6^. However, the mechanisms underlying neuronal injury in AIS are poorly described. Previous studies have shown that ischemic stroke initiates a generalized series of events that occur at the onset of cerebral ischemia^7^. These include cellular bioenergetic failure, oxidative stress, microvascular injury, inflammation, and the eventual necrosis of neuronal, glial and endothelial cells. The time points at which these events occur could be specifically targeted by therapies. However, a number of drugs that have been shown to confer neuroprotective effects on preclinical experiments have failed in a clinical setting^8^. This might be owing to complicated factors involving in treatment of heterogeneous patients^9^. It is widely accepted that this heterogeneity might be the consequence of treatments outside the time frame of efficacy in a real-world AIS clinical setting^10^. Hence, effective drugs are rarely shown to promote neuroprotection and neurorepair of AIS, and the underlying molecular mechanisms of the gap between experimental achievements and clinical solutions remain to be fully explored.

Recently, a new trend in drug development has been to translate the research mode from a single molecule to multiple molecules combined with biological pathways and networks that provides a new method of drug development for complex diseases^11^. The latest evidence shows that different neuropathologies share important commonalities^12^. *N*-methyl-d-aspartate (NMDA) receptors play specific roles in pathological roles that are shared across various neurological and psychiatric disorders^13^. For instance, DJ-1, a Parkinson’s disease gene, is also a key regulator of stroke^14^. Hence, many investigations now focus on identifying new drugs for AIS therapy using therapies that have been proven effective in other diseases.

Network medicine has become increasingly important for identifying novel disease mechanisms and predicting drugs^15^. It provides a network-based approach to elucidate the underlying molecular mechanisms, mainly in terms of disease modules, of disease phenotypes and disease-disease associations^16^,^17^,^18^. To utilize incomplete interactome data for investigating a diseasome (i.e., a disease-disease relationship), Menche *et al*. proposed novel shortest path-based measurement to evaluate overlap between disease modules^19^. Moreover, a novel algorithm was proposed to detect disease modules using incomplete interactome data by taking advantage of the network expansion of disease-related seed genes^20^. A number of studies^21^,^22^ have investigated the disease modules associated with specific disease phenotypes, such as asthma, diabetes and cancer, for which a single disease module would mainly be detected. Recent work has integrated both large-scale electronic medical records and genomic data to detect thousands of novel associations between Mendelian and complex diseases. These studies have revealed that a nondegenerate, phenotypic code links each complex disorder (e.g. cancer, stroke and type 2 diabetes) to a unique collection of Mendelian loci. In addition, recent studies of the molecular subtypes of complex diseases, such as cancer^23^, type 2 diabetes^24^ and psychiatric disorders^25^, have indicated that incorporating the molecular profiles of disease phenotype subtypes that have previously been considered as single disorder would substantially improve our understanding of disease pathophysiology and the outcomes of treatments. Therefore, investigating the disease modules corresponding to the time-frame subtypes of AIS would be a promising research avenue which increase our understanding of the mechanisms underlying the gap between experimental achievements and clinical solutions for AIS.

Here, according to the underlying molecular subtypes of complex diseases and the fragmentation phenomena of disease modules that were extracted from incomplete interactome data^19^, we used a novel disease module detection strategy to identify multi-module structures that might correspond to the disease subtypes of AIS. We first partitioned the entire PPI network into hundreds of topological modules, and then we detected the significant relevant modules of AIS using correlation analysis between the modules and curated AIS-related genes. The curated AIS-related genes were extracted from three phenotype-genotype associations, including the Coremine literature database^26^, OMIM^27^ and DiseaseConnect^28^. The human interactome data were filtered from the String 9.1 PPI database^29^. We used a widely used community detection method (i.e., BGLL) to identify topological modules from the entire human PPI network. After the disease modules associated with AIS were identified by the correlation analysis, we performed a functional analysis of the AIS-related disease modules using gene ontology and pathway enrichment analyses. The key biological functional features of each AIS-related disease module were then mapped to the temporal pathophysiological events that were proposed by Saenger *et al*.^7^. To identify potential drug targets for early time frame subtypes of AIS, we calculated the shortest path distance between AIS disease genes and drug targets. Finally, we confirmed the molecular mechanisms associated with the gap between experimental achievements and clinical solutions for AIS therapies and identified promising novel targets and related drugs for AIS that might confer neuroprotective efficacy.

## Results

### AIS-related disease–gene relationships

To identify AIS-related disease terms, we searched the Medical Subject Headings (MeSH, 2014 version) terminology database on the MeSH Browser website (https://www.nlm.nih.gov/mesh/MBrowser.html) using the key words “stroke” and “infarction”. Using this approach, we ultimately identified (confirmed by the neurobiologists in our author list) 12 AIS-related MeSH headings (Table 1, Table S1).

**Figure 1:**
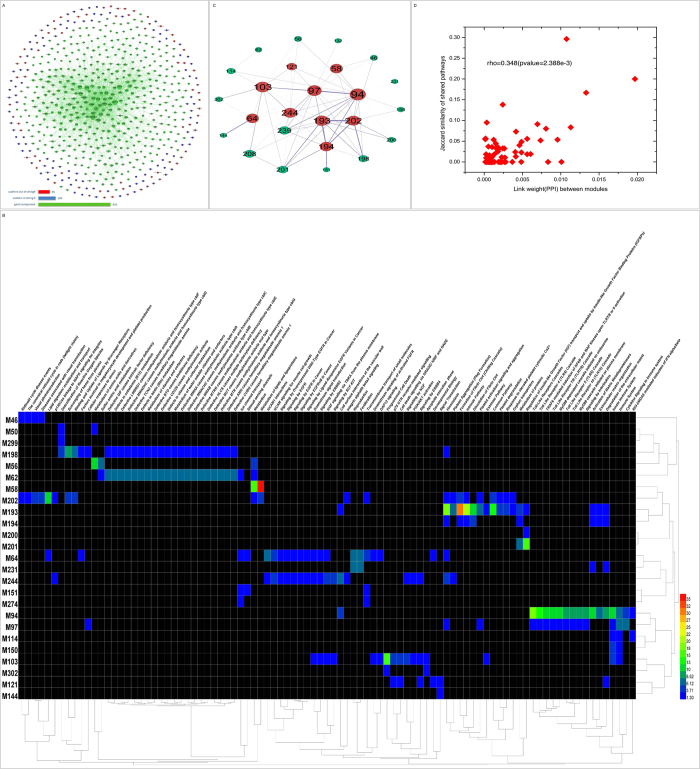
AIS PPI network, intra-module connectivity and heat map of the enriched pathways of the modules. (**A**) AIS PPI network. There were a total of 606 AIS genes. Of these, there were 537 genes (~88.61%) in String 9.1 and 433 genes (~71.45%) in the giant component. (**B**) Heat map of the enriched pathways of the modules with an OR > 2. Pathways are aligned along the x-axis, while modules are aligned along the y-axis. Color code: red, small corrected p value and high enrichment; and green, large corrected p value and low enrichment. (**C**) Intra-module connectivity of the modules with an OR > 2. The red nodes represent the 10 with a large size and higher OR. The green nodes are other modules with an OR > 2. The thickness of the edge is proportional to its weight. Node sizes correspond to the number of edges that cross the node. (**D**) Connection between the edge weight and the pathways shared between the modules. There is a positive correlation (calculated by Spearman correlation) between the edge weight in (**C**) and shared pathways between modules.

Using these 12 MeSH headings as disease keywords, we downloaded 1425 significant disease-gene associations (p < 0.05) in batch mode from the Coremine database^26^. To obtain reliable associations, we performed an extensive check of the PubMed literatures related to the 1425 disease-gene associations, and we confirmed the existence of 1042 (1042/1425 = 73.12%) AIS disease-gene associations involving 606 distinct genes. When we mapped these genes to the human PPI network (see the Supplementary text for our preprocessing methods), only 537 (537/606, 88.61%) genes were kept in the analysis. We visualized the PPI network of the remained AIS-related genes as shown in Fig. 1. In this network, 433 genes (433/606, 71.45%) were included in the giant component (over 12 folds density of the global human PPI network), whereas 69 (69/606, 11.4%) genes were not included, and 104 (104/606, 17.2) genes were scattered in the network. This result is consistent with both the agglomeration of disease proteins in the neighborhood of the observable module and the fragmentation of disease modules^20^ in the context of incomplete interactome data.

To identify the general functional characteristics of AIS-related genes, we performed GO and pathway enrichment analyses of these genes and detected the significant biological processes in which these genes participate (Table S3-S6). In an ischemic area, as a result of a lack of energy, a cascade of neurochemical events occur, ranging from disrupted ion homoeostasis to necrotic and apoptotic cell death^2^. The enrichment analysis results indicated specific biological process features for AIS. For instance, the enriched pathway “Unblocking of NMDA receptor, glutamate binding and activation” (corrected p-value = 2.94 × 10^−4^) is related to glutamate release and calcium transport^30^, whereas the biological processes “Toll-Like Receptor 2 (TLR2) Cascade,” (corrected p-value = 1.759 × 10^−3^) and “Toll-Like Receptor TLR1:TLR2 Cascade” (corrected p-value = 1.759 × 10^−3^) are involved in oxidative stress and cellular necrosis^31^.

### Significant AIS topological modules

To identify the significant disease modules that might correspond to the temporal subtypes of AIS, we partitioned the human PPI network using a widely used community detection algorithm. We confirmed 314 modules of the whole PPI network (see Methods and supplementary text). We obtained 131 modules, including at least one gene from the 537 AIS-related gene list, in which there were 29 modules with significant (Odds Ratio (OR) > 2.0) number of AIS-related genes. To elucidate the inter-module connectivity, we constructed a network using the 29 modules as nodes and links that represented the shared interactions between modules and a heat map that clustered similar modules based on their shared enriched pathways (see Methods, Fig. 1B and C). We found that there were positive correlations (rho = 0.384, p-value = 2.388e-3) between the link weights and shared pathways of modules (Fig. 1D), indicating that the closer two modules are linked, the higher the degree of shared biological processes between them. For example, we found strong interactions between M94 and M97 and many enriched pathways, such as the Toll-Like Receptor Cascades, the Toll-Like Receptor 4 (TLR4) Cascade, and Signaling by Interleukins, are shared between these two modules. We have listed the top 10 topological modules with the highest ORs. These are the modules that were the focus of our further investigations.

**Figure 2:**
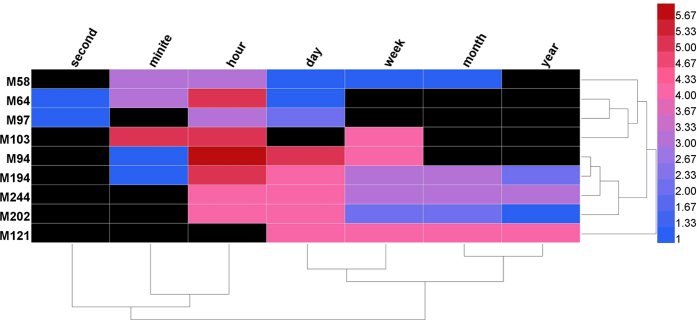
The heatmap of the time frame information of enriched Reactome pathways of ten significant disease modules. The different color types correspond to the number of enriched pathways of the module in the time frame.

Next, we performed a functional analysis of the 10 modules using GO and pathway perspectives, and we found that each of these 10 modules were associated with highly enriched processes. For example, for module M64, the highly enriched GO processes were “ion transport” and “calcium ion transport”, and the enriched pathways were closely relates to the regulation of NMDA receptors, in which Ca^2+^ plays a pivotal role in regulating NMDA receptor activity^32^ (Table S8). Another module, M103, was associated with processes involved in necrosis and programmed cell death. To define the temporal subtypes of AIS, we annotated the pathophysiological events that were involved in the enriched pathways according to the relevant previous studies^7^,^10^,^33^,^34^ (Fig. 2 and Table S2). Our results showed that several modules, such as M64 and M103, contained distinct features that were associated with early molecular events (i.e., occurred within minutes to hours) in AIS. These modules would therefore be appropriate targets for early-stage neuroprotective interventions.

### The molecular network characteristics of AIS-related drug targets

**Figure 3:**
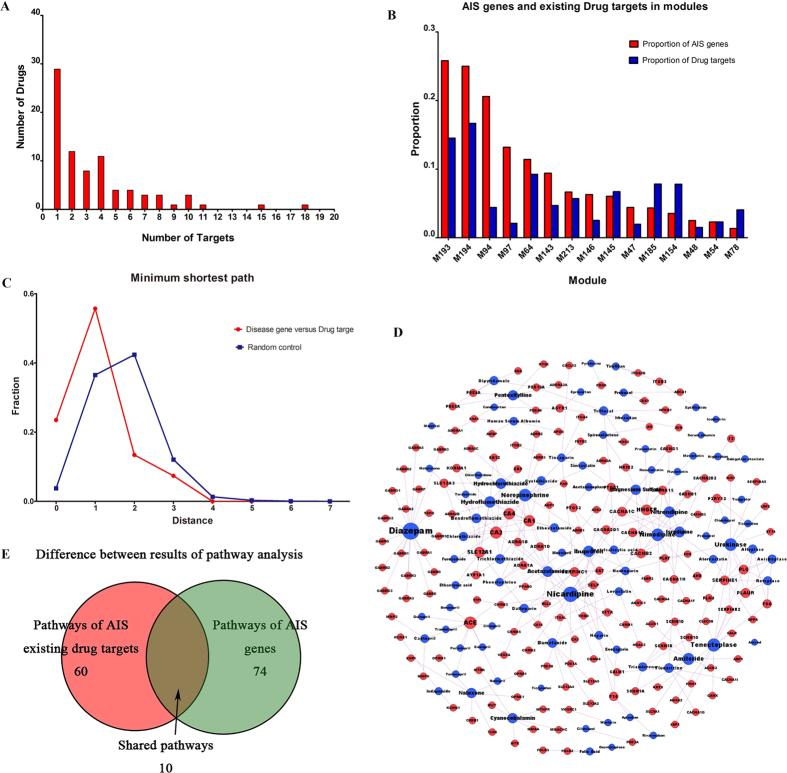
Network of existing AIS drug-targets, statistical analysis of drug targets and analysis of minimum shortest path analysis. (**A**) Analysis of drug-target interactions. Most drugs have several targets. (**B**) Graph showing the statistical analysis of AIS genes and drug targets in modules. For M193, M194 and M64, the proportions of AIS genes and targets are much higher than those of other modules. (**C**) The distribution of minimum shortest paths for the disease data (red) and random control (blue) groups. Enrichment occurs at distances 0 and 1. (**D**) AIS existing drug-target network. The blue nodes represent existing AIS drugs, and the red nodes represent their targets. (**E**) In the pathway analysis showing the results for AIS genes and their existing drug targets, there were 10 overlapping pathways.

We identified 87 drugs from AIS guidelines and their 161 drug targets from the DrugBank database (see Methods, Fig. 3D, Table S2). Only 29 (29/87, 33.3%) of the drugs have a single target (Fig. 3A). A total of 149 (149/161, 92.55%) targets were scattered in the topological modules, and of these, 51 (51/161, 31.68%) targets were included in the 29 significant AIS modules. To measure the molecular interactions between AIS drug targets and genes, we calculated the minimum shortest path lengths between them. This approach was used in a similar previous study^35^(see the Methods section). The results showed that the distances were more enriched at the lower range of distances (i.e., < = 1, Fig. 3C) than the random control. This means that existing AIS drugs regulate diseases by directly targeting disease genes or the neighbors of AIS genes, which might explain the success of current therapies to treat AIS. We also calculated the degrees of AIS drug targets and found that more than 45% of the targets had large degrees (>50) that were statistically higher than those of the entire PPI network (Table S7). These data indicate that shorter distances might be the consequence of the hub effects of AIS-related drug targets.

To further investigate the molecular mechanisms underlying the gap between experimental achievements and clinical solutions with regard to neuroprotection effects, we measured the overlapping pathways both AIS drug targets and genes participating. We identified 84 pathways that were enriched in AIS-related disease genes and 70 pathways that were enriched in AIS drug targets. Only 10 pathways overlapped (P = 2.2 × *e*^−16^), and no neuroprotection-associated pathways were included in the overlapping pathways (Fig. 3E). This result indicates that although current AIS drug targets tend to intervene directly with AIS genes, they are not capable of regulating the neuroprotection-associated pathways that are involved in the early stage pathophysiological events of AIS. These findings suggest that specific and definable molecular network mechanisms underlie the huge gap between experimental achievements and practical clinical solutions (EC-GAP-AIS). A variety of neuroprotective agents have been used in experiments, but these have failed to achieve efficacy in clinical applications^8^. This might be because these agents do not target the correct temporal subtype (i.e. stages) of AIS.

### Novel potential targets and drugs for neuroprotection in AIS

**Figure 4:**
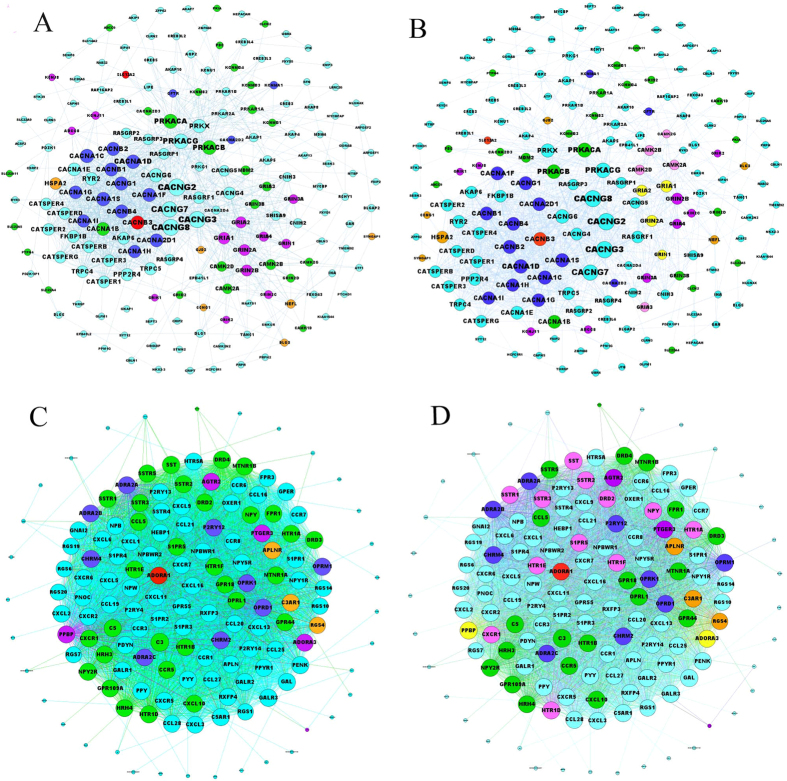
Visualization of M64 and M145. (**A**) and (**B**), visualization of M64. (**C**) and (**D**), visualization of M145. The light blue nodes are not the AIS genes or drug targets in the DrugBank database. The green nodes are the targets of drugs that were not found to be associated with AIS in the DrugBank database. The blue nodes are the targets of AIS drugs that were not associated with AIS genes. The orange-yellow nodes are AIS genes that were not drug targets in the DrugBank database. The purple nodes indicate AIS genes that were targets of drugs that were not associated with AIS in the DrugBank database. The red nodes indicate AIS genes and their drug targets. The pink nodes shown in (**B**) and (**D**) indicate potential targets that were not AIS genes. The yellow nodes in B indicate potential targets that were found to be AIS genes.

Based on the significant disease modules (in particular the modules incorporating early stage pathways) of AIS, we would be able to detect novel drug targets to promote neuroprotection in AIS. Here, we used DrugBank to filter the novel drug targets (those that have not been previously recognized as AIS drug targets) that might act via direct intervention (shortest path length< = 1) with AIS disease genes through the early-stage pathways. First, we identified 5 candidate modules from the significant identified modules that had high ratios of both AIS genes and drug targets in DrugBank. These were M58, M64, M94, M193 and M145. According to the identified functional characteristics (according to GO and pathway analysis), we found that 3 of these modules (i.e., M58, M94 and M193) were not associated with any neuroprotective pathways. These pathways were therefore excluded from further analysis. For example, M58 is closely related to lipid metabolism, and M94 is highly involved in immune responses. It has previously been established that the immune system contributes to the brain damage that is produced by ischemia and that the immune response is involved in all stages of the ischemic cascade^36^. M193 has been shown to be involved in the blood coagulation process. Therefore, these two modules, M64 and M145, have been confirmed as final candidate modules for neuroprotection-promoting drug target discovery. The M64 module (Fig. 4A) includes 184 genes, 21 (21/184, 11.4%) of which are associated with AIS and 57 (57/184 = 31.0%) of which are identified as drug targets in the DrugBank database. We considered the 27 drug targets, which directly or adjacently interact (i.e. the shortest path length < = 1) with the AIS genes, as preliminary candidate drug targets. The results showed that the M64 module is a particular type of molecular module that is incorporated in early-stage AIS events that are associated with neuroprotective processes (Table 2, Table S9). The highly enriched specific pathways, including “Unblocking of NMDA Receptor, Glutamate Binding and Activation”, of M64 (Fig. S2) are also enriched pathways of AIS genes. For example, “Unblocking of NMDA Receptor, Glutamate Binding and Activation” is involved in the initial stage (events occurring within minutes to hours) of AIS. A total of 13 (13/27, 48.1%) preliminary candidate drug targets were shown to be involved in this neuroprotection-related pathway. To further investigate the potential biological functions of these drug targets, we used a recently available proteomics database (called The Human Protein Atlas)^37^ to determine the tissues in which the genes of these targets are significantly expressed. Finally, we confirmed that 9 (9/13, 69.2%) of the targets are expressed in the nervous system, including 4 direct targets, GRIA1, GRIA2, GRIN1and GRIN2A (all of which belong to the glutamate-gated ion channel family), and 5 neighbor targets, CAMK2A, CAMK2B, CAMK2D, CAMK2G and GRIA3 (the first 4 of which belong to the calcium-dependent protein kinase (CAMK) family). These 9 drug targets are potential targets for inducing neuroprotective activity in AIS. Many reports have supported the importance of NMDA receptor subtypes in both neuronal survival and neuronal death^38^, and switches between NMDA receptor subtypes may contribute to reduced plasticity by influencing the binding of active CAMK family members^39^. We obtained 21 clinical drugs, including Primidone and Pethidine, that have targets among the 9 proteins formerly identified in DrugBank (Table S11). Although these 21 drugs are currently used primarily for epilepsy, pain relief and neurodegenerative diseases, searches for the latest drug clinical trials in ClinicalTrials.gov^40^ reveal that recent trials have involved using one or a combination of these drugs (e.g.., Primidone and Pethidine) as treatments for AIS.

Based on our identification of potential targets, we extracted 21 approved drugs, all of which were either antagonists of NMDA receptors or inhibitors of CAMK2. The drugs are used to treat neurological diseases (e.g., seizures, Alzheimer’s disease, and Parkinson’s disease), psychosis (e.g., bipolar disorder, schizophrenia, and delirium), and chronic myelogenous leukemia and to induce and maintain general anesthesia during surgery (Fig. 4A and B).

The M145 module included 149 nodes and was associated with 47 existing drug targets and 9 AIS genes. Of these, 8 (8/47, 17.02%) targets were involved in the highest M145-enriched pathway, “G Alpha (i) Signaling Events,” which is associated with the G-protein coupled receptor (GPCR) (Table S10). The classical signaling mechanism in this pathway involves the inhibition of cyclic adenosine monophosphate (cAMP)-dependent pathways, which are implicated in neuronal cell death^41^. Studies have shown that astrocytes mediate Ca^2+^ signaling by stimulating GPCRs^42^ and that this activity could occur within minutes to hours following stroke^33^. Finally, we identified 14 drug targets for nervous system (Table 3), such as ADORA3, PPBP and CXCR1, in the DrugBank database, in which only 6 targets (i.e., ADORA3, DRD2, HTR1A, HTR1D, HTR1E, and HTR1F) were associated with 25 drugs (i.e. agonists) (Table S11). Of these 25 agonists, several are dopamine receptor agonists or 5-hydroxytryptamine receptors agonists (Fig. 4C and D). Several studies have demonstrated that dopamine receptor agonists can protect against ischemia-induced neurodegeneration^43^ and that 5-hydroxytryptamine receptor agonists can reduce infarct volumes^44^.

## Discussion

Ischemic stroke is a heterogeneous disorder with a variety of clinical symptoms and causes and a diversity of disease subtypes, including CADASIL, lacunar stroke and middle cerebral artery infarction. Early stage intervention has an important impact on prognosis in ischemic stroke patients. However, few such interventions are currently available for use in a clinical setting. Investigations of the molecular network mechanisms (in term of disease modules) that contribute to ischemic stroke are key to identifying the manifestation subtypes and temporal stages of this disorder, and knowledge gained in both of these areas will increase our ability to generate tailored early-stage therapies. We identified over 1000 high-quality AIS disease-gene associations in the PubMed literature database and 29 significant disease modules in the human PPI network. We confirmed the importance of several disease modules, such as M64 and M145, which correspond to the temporal subtypes of AIS and would be highly valuable for early-stage therapies (mainly involving neuroprotection). Furthermore, we identified molecular mechanisms that underlie the gap between experimental achievements and clinical solutions for AIS treatment by measuring the difference between the pathways involving AIS genes and existing drug targets. Based on this investigation, a total of 23 potential targets and dozens of FDA approved drugs (for other disease conditions) were identified. Although the ultimate validation of these novel results will require systematic experimental and clinical studies, we searched the recent literature and found isolated studies that partially validated our results, supporting the reliability of these data. For example, both NMDA receptors and GPCR- related pathways have been identified in M64 disease modules, and related studies have shown that NMDA receptor blockers exert a neuroprotective effect while GPCR agonists are associated with cell apoptosis^45^,^46^,^47^. Otherwise, the activation of NMDA receptors and GPCRs contributes to Ca^2+^ overload^10^,^42^, which occurs within minutes of stroke onset^33^.

NMDA receptors are involved in a variety of neurological and psychiatric diseases^48^,^49^. For instance, NMDA receptors initiate neuronal death and the neurodegenerative processes in Alzheimer’s disease^50^. These antagonists can be used to treat cognitive dysfunctions, such as dementia^51^. Many successful experiments have demonstrated that NMDA antagonists confer a neuroprotective effect against the progression of ischemic stroke^52^,^53^, and GRIN2A is increased immediately after the onset of stroke symptoms^7^. Due to the shared molecular and phenotype features associated with various neurological diseases, the drugs used to treat the other similar neurological diseases may also create an effective neuroprotective effect against AIS. Of the candidate drugs identified in this study, felbamate and methylphenobarbital, which are used to treat epilepsy, and memantine, which is used to treat Alzheimer’s disease, may also be effectively used to treat AIS.

The clinical failure of NMDA receptor antagonists is partly because of the time frame in which they are applied. Whereas in experimental models, the onset of ischemia and reperfusion can be precisely defined, this is not possible in a clinical setting, and treatment might therefore be delayed before the presence of the disease is realized^10^. In a clinical setting, the timing of treatment might be delayed until the patient becomes aware of the cerebral ischemia. The time points at which these clinical drugs are used are often outside the window of opportunity to act as an effective neuroprotective treatment^7^.

In addition, the significant pathways associated with M145 and G alpha (i) signaling events exert a neuroprotective effect by inhibiting the cAMP-dependent pathway, which inhibits apoptosis^41^. This pathway also participates in the initial stage of stroke by regulating Ca^2+^signaling. This pathway is also related to stem cell functions. The role played by G alpha (i) signaling in pluripotent stem cells is largely unknown, but it involves the maintenance of pluripotency and the directed differentiation of human embryonic stem cells^54^. Histone modifications are thought to play certain key roles in cell reprogramming^55^, which plays a crucial role in establishing nuclear totipotency during normal development^56^. In the current study, the candidate ADORA3 is targeted by the antagonist aminophylline. The description of aminophylline in the DrugBank database indicates that aminophylline can modify histones by activating histone deacetylase. Hence, ADORA3 could be involved at a specific point during reprogramming. In addition, reprogramming may represent an endogenous process that protects the brain against further injury^57^. Therefore, regulating G alpha (i) signaling events might be a new avenue for further studies of AIS treatments.

Moreover, ADORA3 agonists can regulate the extracellular matrix (ECM) to protect against neuronal death^58^. During the initial stage of stroke, the upregulation of matrix metalloproteinases (MMPs) damages the blood brain barrier (BBB) by degrading the neurovascular matrix and thereby contributing to neuronal death. However, MMPs also promote angiogenesis during neurovascular repair phases^34^. Published evidence has shown that ADORA3 agonists can increase the secretion of MMPs^59^.These agonists may therefore promote effective neuroprotection and neurorepair in AIS.

## Methods

### Disease-gene associations

To identify AIS-related disease terms, we searched the Medical Subject Headings (MeSH, 2014 version) terminology database using the key words “stroke” and “infarction” at the MeSH Browser website (https://www.nlm.nih.gov/mesh/MBrowser.html). Following this search, the neurobiologists in our author list ultimately confirmed 12 AIS-related MeSH headings. Using these 12 MeSH headings as disease keywords, we downloaded 1425 significant disease-gene associations (p < 0.05) in batch mode from the Coremine database.

We searched the PubMed literature database for these disease-gene associations, and we confirmed 1042 disease-gene associations by reading more than 4500 PubMed articles.

### AIS existing drugs-targets associations

In recent years, the American College of Cardiology (ACC) and the American Heart Association (AHA) have classified ischemic stroke as a atherosclerotic cardiovascular disease events^60^. We obtained known AIS drugs from the guideline designed by AHA and American Stroke Association (ASA) professionals^61^,^62^. Comprehensive information was obtained from the DrugBank database about these drugs^63^.

### Detection of PPI topological modules

Community structures are widely distributed in complex networks. Each community is comprised of nodes that densely connect its members and are sparsely connected with the nodes in other modules^64^. A variety of community detection algorithms can be used to identify the topological modules in a large-scale network. We used a widely used algorithm called BGLL^65^, which is based on modularity evaluation, to obtain the topological modules of the full PPI network. Because BGLL yielded some modules with very large (e.g. several thousand) member nodes, we iteratively divided the modules using BGLL, resulting in topological modules that contain between 5 and 400 member nodes. However, different community detection algorithms would exactly obtain different results. Therefore, to validate the potential influence of our results, we performed another analysis using a network partition method based on the NMF^66^ algorithm to obtain a similar number of communities for comparison (see Supplementary text).

### GO enrichment analysis

GO enrichment analysis defines gene groups based on the categories in the Gene Ontology database^67^. Using the plugin BiNGO 2.44 of Cytoscape 2.8.2 68,69, an open-source software platform for visualizing molecular interaction networks, we analyzed the disease genes, existing drug targets, and proteins in the modules that we previously characterized.

### Reactome pathway analysis

Pathway analysis has become an important method for gaining insight into the biological functions underlying genes and proteins^70^. The Reactome database^71^ is a manually curated, open-source and open-data resource of human pathways. We obtained the enriched Reactome pathways using the online software program KOBAS2.0^72^.

### Intra-module connectivity

Next, we extracted the outgoing edges between the nodes in the different modules with an OR > 2. A link was documented when two modules were found to have PPI interactions between each other. For example, if there are *c* nodes in module M1, *d* nodes in M2, and *e* edges between M1 and M2, the weight of the edge M1-M2 would be:


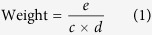


The heavier the weight, the closer the interaction between the two modules. Using the topological connectivity, we were able identify biological connections using the functional analysis^73^.

### The shortest paths between drug targets and seed genes

Shortest paths are significant topological and statistical quantities that are used to analyze social and biological networks. The most outstanding example of the use of these quantities is the well-known small world property of many complex networks^18^. We used Dijkstra’s algorithm to identify the shortest path lengths between AIS drug targets and the genes of interest confirmed in this study^74^. To obtain random controls for the target-genes, we generated 100 independent randomized samples using the PPI network. Significant differences were calculated using t-tests (see supplementary text).

## Additional Information

**How to cite this article**: Wang, Y. *et al*. Network-Based Approach to Identify Potential Targets and Drugs that Promote Neuroprotection and Neurorepair in Acute Ischemic Stroke. *Sci. Rep.* **7**, 40137; doi: 10.1038/srep40137 (2017).

**Publisher's note:** Springer Nature remains neutral with regard to jurisdictional claims in published maps and institutional affiliations.

## Supplementary Material

Supplementary Dataset 1

Supplementary Dataset 2

Supplementary Dataset 3

Supplementary Dataset 4

Supplementary Dataset 5

Supplementary Dataset 6

Supplementary Dataset 7

Supplementary Information

## Figures and Tables

**Table 1:** Acute ischemic stroke–related MeSH headings.

ID	MeSH headings	Scope Note
D002544	Cerebral Infarction	The formation of an area of necrosis in the cerebrum caused by an insufficiency of arterial or venous blood flow. Infarcts of the cerebrum are generally classified by hemisphere, lobe, arterial distribution, and etiology.
D020767	Intracranial Thrombosis	Formation or presence of a blood clot in a blood vessel within the SKULL. Intracranial thrombosis can lead to thrombotic occlusions and brain infarction.
D020766	Intracranial Embolism	Blocking of a blood vessel in the skull by an embolus which can be a blood clot or other undissolved material in the blood stream.
D002542	Intracranial Embolism and Thrombosis	Embolism or thrombosis involving blood vessels which supply intracranial structures. Emboli may originate from extracranial or intracranial sources.
D020243	Infarction, Anterior Cerebral Artery	Necrosis occurring in the anterior cerebral artery system, including branches such as Heubner’s artery. Infarction in anterior cerebral artery usually results in sensory and motor impairment in the lower body.
D020244	Infarction, Middle Cerebral Artery	Necrosis occurring in the middle cerebral artery distribution system which brings blood to the entire lateral aspects of each cerebral hemisphere. Clinical signs include impaired cognition; aphasia; agraphia; weak and numbness in the face and arms, contralaterally or bilaterally depending on the infarction.
D020762	Infarction, Posterior Cerebral Artery	Necrosis induced by ischemia in posterior cerebral artery distribution system which supplies portions of the brain stem, thalamus, temporal and occipital lobe. Clinical features include olfaction disorders and visual problems.
D002546	Ischemic Attack, Transient	Brief reversible episodes of focal, nonconvulsive ischemic dysfunction of the brain having a duration of less than 24 hours, and usually less than 1 hour, caused by transient thrombotic or embolic vessel occlusion or stenosis.
D046589	CADASIL	CADASIL is an acronym for Cerebral Autosomal Dominant Arteriopathy with Subcortical Infarcts and Leukoencephalopathy.
D020925	Hypoxia-Ischemia, Brain	A disorder characterized by a reduction of oxygen in the blood combined with reduced blood flow to brain from a localized obstruction of a cerebral artery or from systemic hypoperfusion. Prolonged hypoxia-ischemia is associated with TIA; brain infarction and other conditions.
D059409	Stroke, Lacunar	Stroke caused by lacunar infarction or other small vessel diseases of the brain. It features hemiparesis, hemisensory, or hemisensory motor loss.
D002545	Brain Ischemia	Localized reduction of blood flow to brain tissue due to arterial obstruction or systemic hypoperfusion. This frequently occurs in conjunction with brain hypoxia. Prolonged ischemia is associated with brain infarction.

**Table 2:** Pathways enriched for M64.

Pathway	PV	CPV	Time frame
Activation of NMDA receptor upon glutamate binding and postsynaptic events	1.73E-20	1.36E-18	mh
Glutamate Binding, Activation of AMPA Receptors and Synaptic Plasticity	3.54E-20	2.63E-18	smh
Trafficking of AMPA receptors	3.54E-20	2.63E-18	hd
Unblocking of NMDA receptor, glutamate binding and activation	4.17E-19	2.99E-17	mh
Depolarization of the Presynaptic Terminal Triggers the Opening of Calcium Channels	1.22E-15	6.96E-14	mh
Post-NMDA receptor activation events	1.62E-15	8.97E-14	mh
Ras activation upon Ca2+ influx through NMDA receptor	4.55E-14	2.27E-12	mh
CREB phosphorylation through the activation of CaMKII	5.85E-13	2.78E-11	mh
CREB phosphorylation through the activation of Ras	1.72E-12	7.69E-11	mh
Integration of energy metabolism	2.59E-11	1.01E-09	m
NCAM1 interactions	8.29E-10	2.84E-08	h
Rap1 signaling	6.49E-09	1.97E-07	hd
NCAM signaling for neurite out-growth	7.11E-08	1.84E-06	dwMy
PKA activation	2.28E-07	5.62E-06	mh
PKA activation in glucagon signaling	3.06E-07	7.43E-06	mh

Here, we describe 15 highly enriched pathways of M64. Most of these pathways are induced during the early stage of ischemic stroke (within minutes to hours (mh)). Two pathways are involved from hours to days (hd), and the pathway “NCAM signaling for neurite out-growth” can last for days, weeks, months or years (dwMy) (Table S6).

**Table 3:** Spatial localization of potential acute ischemic stroke targets in M64 and M145.

Tissue	Cerebral Cortex				Hippocampus	
Cell	Endothelial cells	Glial cells	Neuronal cells	Neuropil	Glial cells	Neuronal cells
M64	GRIA2	CAMK2D	CAMK2A	CAMK2A	GRIA3	CAMK2A
	CAMK2A	CAMK2G	CAMK2B		GRIN1	CAMK2B
		GRIN1	CAMK2D	CAMK2B		CAMK2D
	GRIA3		CAMK2G	CAMK2D		CAMK2G
	GRIN2A	GRIN2A		CAMK2G		GRIA1
			GRIA1	GRIA2		
			GRIA2	GRIA3		GRIA2
			GRIA3	GRIN1		GRIN1
			GRIN1	GRIN2A		
			GRIN2A			
M145	DRD2	HTR1D	CXCR1	ADORA3	DRD2	CXCR1
		HTR1E			HTR1D	
	HTR1A		HTR1A	DRD2		HTR1D
	HTR1E		HTR1D	HTR1A	HTR1E	HTR1E
			HTR1E	HTR1D		
			NPY	HTR1E		NPY
			PPBP	HTR1F NPY		
			SST	S1PR5		SSTR3
			SSTR2	SSTR1		
			SSTR3	SSTR2		
				SSTR3		

We identified 9 potential targets in M64 and 14 potential targets in M145.
