# Supplementary material for: Network-Based Approach to Identify Potential Targets and Drugs that Promote Neuroprotection and Neurorepair in Acute Ischemic Stroke
Source: Sci Rep. 2017 Jan 5;7:40137. doi: 10.1038/srep40137 (PMC5215297; doi:10.1038/srep40137)
Supplement: Supplementary Information [file srep40137-s36.pdf]

Network-Based Approach to Identify Potential Targets and Drugs that Promote Neuroprotection and Neurorepair in Acute Ischemic Stroke

Yiwei Wang[4+], Hailong Liu[1+], Yongzhong Lin[5+], Guangming Liu[6], Hongwei Chu[4], Pengyao Zhao[6], Xiaohan Yang[4], Tiezheng Zheng[4], Ming Fan[7], Xuezhong Zhou[6,*], Jun Meng[8,*], Changkai Sun[1,2,3,4*]

[+]These authors contributed equally to this work.

[1]Department of Biomedical Engineering, Faculty of Electronic Information and Electrical Engineering, Dalian University of Technology, Dalian 116024, China

[2]Research Center for the Control Engineering of Translational Precision Medicine, Dalian University of Technology, Dalian 116024, China

[3]State Key Laboratory of Fine Chemicals, Dalian R&D Center for Stem Cell and Tissue Engineering, Dalian University of Technology, Dalian 116024, China

[4]Liaoning Provincial Key Laboratory of Cerebral Diseases, Institute for Brain Disorders, Dalian Medical University, Dalian 116044, China

[5]Department of Neurology, the Second Affiliated Hospital of Dalian Medical University, Dalian 116027, China

[6]School of Computer and Information Technology and Beijing Key Lab of Traffic Data Analysis and Mining, Beijing Jiaotong University, Beijing 100044, China

[7]Institute of Basic Medical Sciences, Academy of Military Medical Sciences, Beijing 100850, China

[8]College of Electrical engineering, Zhejiang University, Hangzhou 310027, China

***Corresponding authors:**

Changkai Sun: cksun110@vip.sina.com

Xuezhong Zhou: xzzhou@bjtu.edu.cn

Jun Meng: junmeng@zju.edu.cn

1. Basic datasets compilation and preprocessing

The datasets used in our work include AIS MeSH headings, AIS disease-gene relationships, human protein-protein interactions and drug-target relationships. We will introduce the main compilation and preprocessing tasks in the following sections.

1.1Disease-gene relationships

To identify the AIS related disease terms, we searched the Medical Subject Headings (MeSH, 2014 version) terminology database using the key words “stroke” and “infarction” on the MeSH Browser website (https://www.nlm.nih.gov/mesh/MBrowser.html) and ultimately confirmed (by the neurobiologists in our author list) 12 AIS related MeSH headings (**Table S1**)

With these 12 MeSH headings as disease keywords, we downloaded 1425 significant disease-gene associations ($p<0.05$)in batch mode from Coremine database[1].

1.2 Curation of AIS disease-gene associations

Since we have downloaded all the related PubMed identifiers relevant to each disease-gene association, we manually checked the reliability of each disease-gene association by the several following key steps. 1) We ranked the related PubMed literatures of disease-gene associations in descending order by their publication date; 2) For each disease-gene associations, we manually checked each of the related PubMed literatures from the latest one. 3)If we find there are clear declarations in the literatures to indicate the disease-gene association is exactly positive, then the corresponding disease-gene association would be considered as reliable. 4) Otherwise, if we find clear indications to show that the disease-gene association is negative, then we would annotate the corresponding disease-gene association as false one. 5) If we cannot confirm from the current literature, we would go next literature, which is published earlier than the current one. When we go through all the PubMed literatures of a disease-gene association, but could not confirm whether it is true or not, we would exclude it from our final results as well. There are more than 4500 PubMed literatures been readed.

From PubMed literatures, we found out the sentences supporting or objecting to the disease-gene associations. If there is evidence supporting a disease-gene association, it will be a real relationship and gene in it is a credible gene. Finally, 1042 (1042/1425 = 73.12%) real relationships were confirmed

containing 606 distinct genes. To evaluate quality of our data, we checked two other well-known disease-gene association databases: OMIM[2](http://www.omim.org/) and DiseaseConnect[3] (http://disease-connect.org/). We find that there are9 AIS genotype-phenotype relationships containing 6 genes in the OMIM database and 3 disease–gene relationships with 2 genes in the DiseaseConnect database. It is interesting that all of these genes are included in our AIS disease-gene associations curated from CoreMine database. (**Data S1**)

1.3 Human protein-protein interactions

Although with numerous curation efforts, human protein-protein interactome(PPI) data is incomplete. However, there are several integrated high quality PPI databases like STRING 9.1 database[4](http://www.string-db.org/)that we could utilize. STRING 9 database is a comprehensive PPI data source developed by integrating various data sources of experimental, predicted and transferred interactions, together with interactions obtained through text mining.It conteins about 5 million proteins and >200 million interactions. There is a confidence level of each interaction. The interactions whose score≥700 have high confidence or better. We filtered to obtain the high quality human PPI records by the weighted score≥700 and finally obtained 218,409 PPI records with 15,551 proteins.

1.4 AIS existing drugs-targets associations

In recent years, the American College of Cardiology (ACC) and the American Heart Association (AHA) have classified ischemic stroke into the study of atherosclerotic cardiovascular disease events[5]. We obtained 87 known AIS drugs from the guideline designed by AHA and American Stroke Association (ASA) professionals[6, 7]. Comprehensive information about these drugs was obtained from the DrugBank database[8](http://www.drugbank.ca/). And there are totally 161 targets of these drugs. (Table S2)

2 Detection of PPI topological modules

Community structures are widely existed in complex network, for each community it comprises of nodes that are densely connected among its members and sparsely connected with nodes in other modules[9]. There are various community detection algorithms that can be used for identifying the topological modules from large-scale network. We used the widely used algorithm (called BGLL) [10], which is based on modularity evaluation, to obtain the

topological modules of the whole PPI network. Because BGLL would get some modules with very large (e.g. several thousand) member nodes, we iteratively divided the modules by BGLL and finally obtained the topological modules with the number of member nodes between 5 and 400. However, different community detection algorithms would exactly obtain different results. Therefore, to validate the possible influence to our results, we performed another network partition method based on NMF algorithm to get the similar number of communities for comparison.

We identified 301 modules from String 9.1 PPI network. We assumed that if the module results are similar, we could find similar communities from another module set for a given module in one module set. Therefore, we calculated the Jaccard similarity between each pairs of modules derived from two module sets. Then we calculated the module similarity distribution. The similarities of 78.095% modules are more than 0.6(Figure S1). So there is no siginificant differences between these two therapies.

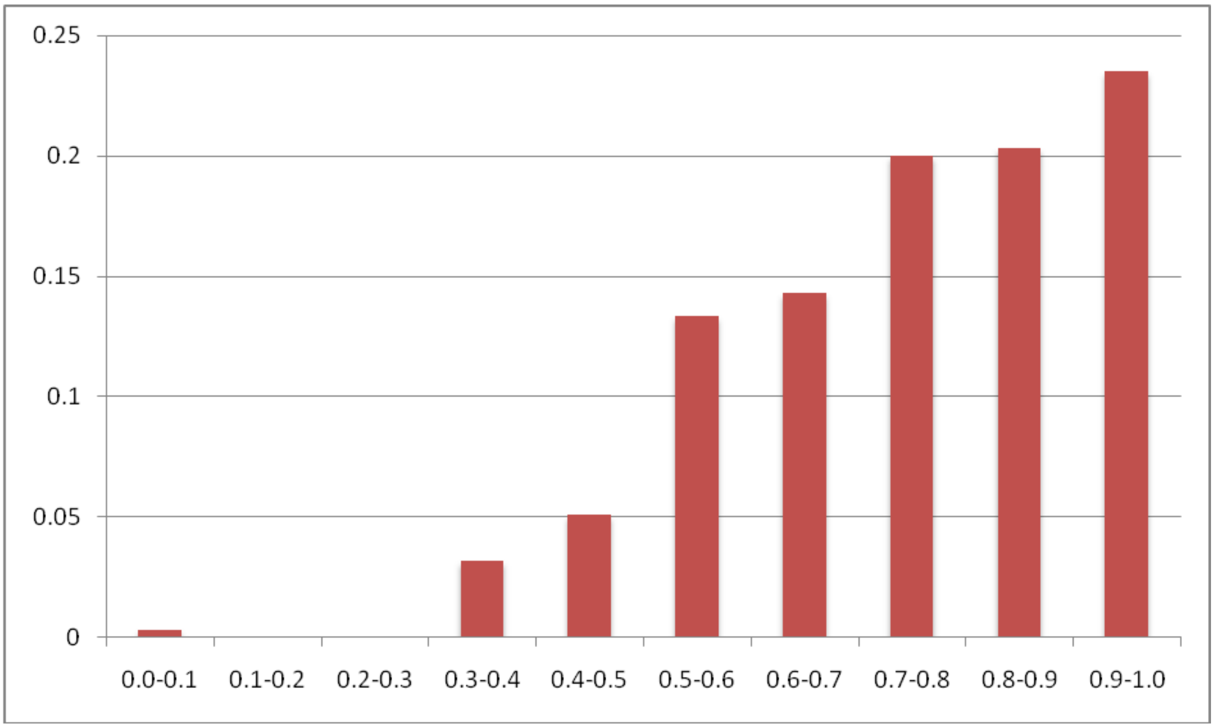

Figure S1.Jaccard similarity between different modules results.

We counted the number of disease genes in each module and calculate OR (Odds Ratio). Assume that there are *A* disease genes in the whole PPI metwork and distribute in many modules in which there totally are *M* nodes. If there are *a* disease genes in a module which has *m* nodes, then the OR of this module is:

$$OR = \frac{a/(m-a)}{A/(M-A)}, (1)$$

There are 71 modules whose OR>1.0 and 29 modules whose OR>2.0. In the **Data S2**, there are the nodes information of the 29 modules.

3 GO enrichment analysis

The GeneOntology database[11] provides three different types of annotations for proteins: biological process, molecular function, and cellular component. GO enrichment analysis can define gene groups based on the categories in the GeneOntology database[12].With the plugin BiNGO 2.44 of Cytoscape 2.8.2[13, 14], an open-source software platform for visualizing molecular interaction networks, we analyzed the disease genes, existing drug targets, and proteins in the modules. And the statistical test is Hypergeometric test and Benjamini & Hochberg False Discovery Rate (FDR) correction to gain a corrected P-value. The significance level is 0.05.

In the result of AIS credible genes, there are 1392 Biological process terms, 216 Molecular function terms and 108 Cellular component terms. Here we list part of them. **(Table S3-S5, Data S3)**

In the GO enrichment analysis result of AIS existing durg targets, there are 860 Biological process terms, 213 Molecular function terms and 88 Cellular component terms. **(Data S4)**

The GO analysis result of 29 modules (OR>2) are showed in **Data S5.**

4 Pathway enrichment analysis

Pathway analysis has become a vitally important method for gaining insight into the underlying biological functions of genes and proteins. The Reactome database is a manually curated open-source and open-data resource of human pathways. We obtained the enriched Reactome pathways using the KOBAS2.0 online software[15](http://kobas.cbi.pku.edu.cn/home.do). In the pathway analysis result of AIS credible genes, there are 84 enriched pathways. All of them are listed in **Table S6.** And enriched pathways of AIS existing drug targets（Data S6）and 29 modules(OR >2) are showed in **Data S7**.

5 Shortest paths between drug targets and seed genes

The shortest paths are a significant topological statistical quantity used for the analysis of social and biological networks. The most outstanding example of its use is likely to be the well-known small world property of many complex networks16. We used the Dijkstra's algorithm to find the shortest path lengths between AIS drug targets and credible genes17. To obtain random controls for the target-gene, we generated 100 independent randomized samples in the PPI network. Significant difference was calculated statistically using t-test analysis. In PPI network, the hubs is not suitable for AIS treatment18. However the shortest paths analysis result showed that the distances between AIS drug targets and credible genes are enriched at the low range distances (i.e. <=1). This means that existing AIS drugs regulate disease by targeting disease genes directly or neighbors of AIS genes. Otherwise we caculated the degrees of AIS related drug targets in which degrees of more than 45% targets are bigger than 50. It is statistically higher than those of the whole PPI network (Table S7).

6 Analysis of potential targets

6.1 Potential targets in M64

In M64, there are 184 nodes containing 21 AIS genes and 57 drug targets in DrugBank database. Proteins in it are enriched in 81 Reactome pathways. Here we list the top 20 pathways containing 7 pathways shared with the result of AIS credible genes (**Table S8** and **Table S9**). Between these 7 pathways, there is an including and included relationship **(Figure S2)**.With the pathway "Unblocking of NMDA receptor, glutamate binding and activation", we identified 9 potential targets whose lenth from AIS genes is ≤1.

6.2 Analysis of M145

In M64, there are 145 nodes containing 9 AIS genes and 47 drug targets in Drugbank. Proteins in it are enriched in 28 Reactome pathways. And 20 pathways are shared with the result of AIS credible genes (**Table S10**). The most enriched pathway "G Alpha (i) Signaling Events" is at the bottom of the pathway tree. There are 14 potential targets on this pathway. The distances between them and AIS genes are 0 or 1.

7 Potential Drugs

With the potential targets in M64 and M145, we screened out some drugs from DrugBank database. With the analysis of M64, we found out 21 potential drugs. Otherwise, 25 potential drugs were based on the analysis of M145. All of them are showed in **Table S11**.

Table S1. Acute ischemic stroke–related MeSH headings.

Unique ID	Unidentified MeSH headings	Identified MeSH headings	Scope Note
D002544	Cerebral Infarction	Cerebral Infarction	The formation of an area of necrosis in the cerebrum caused by an insufficiency of arterial or venous blood flow. Infarcts of the cerebrum are generally classified by hemisphere (i.e., left vs. right), lobe (e.g., frontal lobe infarction), arterial distribution (e.g., infarction, anterior cerebral artery), and etiology (e.g., embolic infarction).
D020767	Intracranial Thrombosis	Intracranial Thrombosis	Formation or presence of a blood clot (thrombus) in a blood vessel within the SKULL. Intracranial thrombosis can lead to thrombotic occlusions and brain infarction. The majority of the thrombotic occlusions are associated with arterosclerosis.
D020766	Intracranial Embolism	Intracranial Embolism	Blocking of a blood vessel in the skull by an embolus which can be a blood clot (thrombus) or other undissolved material in the blood stream. Most emboli are of cardiac origin and are associated with heart diseases. Other non-cardiac sources of emboli are usually associated with vascular diseases.

D002542	Intracranial Embolism and Thrombosis	Intracranial Embolism and Thrombosis	Embolism or thrombosis involving blood vessels which supply intracranial structures. Emboli may originate from extracranial or intracranial sources. Thrombosis may occur in arterial or venous structures.
D020243	Infarction, Anterior Cerebral Artery	Infarction, Anterior Cerebral Artery	necrosis occurring in the anterior cerebral artery system, including branches such as Heubner's artery. These arteries supply blood to the medial and superior parts of the cerebral hemisphere, Infarction in the anterior cerebral artery usually results in sensory and motor impairment in the lower body.
D020244	Infarction, Middle Cerebral Artery	Infarction, Middle Cerebral Artery	necrosis occurring in the middle cerebral artery distribution system which brings blood to the entire lateral aspects of each cerebral hemisphere. Clinical signs include impaired cognition; aphasia; agraphia; weak and numbness in the face and arms, contralaterally or bilaterally depending on the infarction.
D020762	Infarction, Posterior Cerebral Artery	Infarction, Posterior Cerebral Artery	Necrosis induced by ischemia in the posterior cerebral artery distribution system which supplies portions of the brain stem; the thalamus; temporal lobe, and occipital lobe. Depending on the size and location of infarction, clinical features include olfaction disorders and visual problems.
D002546	Ischemic Attack, Transient	Ischemic Attack, Transient	Brief reversible episodes of focal, nonconvulsive ischemic dysfunction of the brain having a duration of less than 24 hours, and usually less

			than one hour, caused by transient thrombotic or embolic blood vessel occlusion or stenosis. Events may be classified by arterial distribution, temporal pattern, or etiology
D046589	CADASIL	CADASIL	A familial, cerebral arteriopathy mapped to chromosome 19q12, and characterized by the presence of granular deposits in small cerebral arteries producing ischemic stroke; pseudobulbar palsy; and multiple subcortical infarcts. CADASIL is an acronym for Cerebral Autosomal Dominant Arteriopathy with Subcortical Infarcts and Leukoencephalopathy. CADASIL differs from binswanger disease by the presence of migraine with aura and usually by the lack of history of arterial hypertension.
D020925	Hypoxia-Ischemia, Brain	Hypoxia-Ischemia, Brain	A disorder characterized by a reduction of oxygen in the blood combined with reduced blood flow (ischemia) to the brain from a localized obstruction of a cerebral artery or from systemic hypoperfusion. Prolonged hypoxia-ischemia is associated with ischemic attack, transient; brain infarction; brain edema; coma; and other conditions.
D059409	Stroke, Lacunar	Stroke, Lacunar	Stroke caused by lacunar infarction or other small vessel diseases of the brain. It features hemiparesis, hemisensory, or hemisensory motor loss.

D002545	Brain Ischemia	Brain Ischemia	Localized reduction of blood flow to brain tissue due to arterial obstruction or systemic hypoperfusion. This frequently occurs in conjunction with brain hypoxia. Prolonged ischemia is associated with brain infarction.
D020521	Stroke		

Table S2. AIS existing Drug-target associations. There are 292 associations between 87drugs and161 targets.

**DrugID**	**Drug name**	**Groups**	**Target**
**DB00009**	Alteplase	Approved	SERPINE1
**DB00009**	Alteplase	Approved	PLG
**DB00009**	Alteplase	Approved	FGA
**DB00009**	Alteplase	Approved	PLAUR
**DB00013**	Urokinase	Approved, Investigational, Withdrawn	SERPINE1
**DB00013**	Urokinase	Approved, Investigational, Withdrawn	PLG
**DB00013**	Urokinase	Approved, Investigational, Withdrawn	PLAUR
**DB00013**	Urokinase	Approved, Investigational, Withdrawn	PLAU
**DB00013**	Urokinase	Approved, Investigational, Withdrawn	LRP2
**DB00013**	Urokinase	Approved, Investigational, Withdrawn	SERPINB2
**DB00013**	Urokinase	Approved, Investigational, Withdrawn	PLAT
**DB00013**	Urokinase	Approved, Investigational, Withdrawn	ST14
**DB00013**	Urokinase	Approved, Investigational, Withdrawn	SERPINA5
**DB00013**	Urokinase	Approved, Investigational, Withdrawn	NID1
**DB00015**	Reteplase	Approved	SERPINE1
**DB00015**	Reteplase	Approved	PLG
**DB00015**	Reteplase	Approved	FGA

**DB00015**	Reteplase	Approved	PLAUR
**DB00029**	Anistreplase	Approved	SERPINE1
**DB00029**	Anistreplase	Approved	PLG
**DB00029**	Anistreplase	Approved	FGA
**DB00029**	Anistreplase	Approved	PLAUR
**DB00031**	Tenecteplase	Approved	SERPINE1
**DB00031**	Tenecteplase	Approved	PLG
**DB00031**	Tenecteplase	Approved	CANX
**DB00031**	Tenecteplase	Approved	FGA
**DB00031**	Tenecteplase	Approved	PLAUR
**DB00031**	Tenecteplase	Approved	SERPINB2
**DB00031**	Tenecteplase	Approved	ANXA2
**DB00031**	Tenecteplase	Approved	CLEC3B
**DB00031**	Tenecteplase	Approved	KRT8
**DB00031**	Tenecteplase	Approved	CALR
**DB00031**	Tenecteplase	Approved	LRP1
**DB00062**	Human Serum Albumin	Approved	AMBP
**DB00062**	Human Serum Albumin	Approved	APOE
**DB00062**	Human Serum Albumin	Approved	SAA1
**DB00063**	Eptifibatide	Approved, Investigational	ITGB3
**DB00115**	Cyanocobalamin	Approved, Nutraceutical	MMAA
**DB00115**	Cyanocobalamin	Approved, Nutraceutical	MTR
**DB00115**	Cyanocobalamin	Approved, Nutraceutical	MTRR
**DB00115**	Cyanocobalamin	Approved, Nutraceutical	MUT
**DB00115**	Cyanocobalamin	Approved, Nutraceutical	MMACHC
**DB00115**	Cyanocobalamin	Approved, Nutraceutical	MTHFR

**DB00158**	Folic Acid	Approved, Nutraceutical	FOLR2
**DB00158**	Folic Acid	Approved, Nutraceutical	FOLR3
**DB00165**	Pyridoxine	Approved, Nutraceutical	PDXK
**DB00175**	Pravastatin	Approved	HMGCR
**DB00178**	Ramipril	Approved	ACE
**DB00208**	Ticlopidine	Approved	P2RY12
**DB00214**	Torasemide	Approved	SLC12A1
**DB00227**	Lovastatin	Approved, Investigational	ITGAL
**DB00227**	Lovastatin	Approved, Investigational	HMGCR
**DB00227**	Lovastatin	Approved, Investigational	HDAC2
**DB00270**	Isradipine	Approved	CACNA2D1
**DB00270**	Isradipine	Approved	CACNA1H
**DB00270**	Isradipine	Approved	CACNA1C
**DB00270**	Isradipine	Approved	CACNB2
**DB00270**	Isradipine	Approved	CACNA1S
**DB00270**	Isradipine	Approved	CACNA1D
**DB00270**	Isradipine	Approved	CACNA2D2
**DB00278**	Argatroban	Approved, Investigational	F2
**DB00310**	Chlorthalidone	Approved	SLC12A1
**DB00316**	Acetaminophen	Approved	PTGS2
**DB00316**	Acetaminophen	Approved	PTGS1
**DB00368**	Norepinephrine	Approved	ADRB1
**DB00368**	Norepinephrine	Approved	ADRA2A
**DB00368**	Norepinephrine	Approved	ADRA2C
**DB00368**	Norepinephrine	Approved	ADRB3
**DB00368**	Norepinephrine	Approved	ADRB2
**DB00368**	Norepinephrine	Approved	ADRA1D
**DB00368**	Norepinephrine	Approved	ADRA2B
**DB00368**	Norepinephrine	Approved	ADRA1B

**DB00368**	Norepinephrine	Approved	PAH
**DB00368**	Norepinephrine	Approved	ADRA1A
**DB00384**	Triamterene	Approved	SCNN1B
**DB00384**	Triamterene	Approved	SCNN1A
**DB00384**	Triamterene	Approved	SCNN1D
**DB00384**	Triamterene	Approved	SCNN1G
**DB00388**	Phenylephrine	Approved	ADRA1D
**DB00388**	Phenylephrine	Approved	ADRA1B
**DB00388**	Phenylephrine	Approved	ADRA1A
**DB00393**	Nimodipine	Approved	CACNB1
**DB00393**	Nimodipine	Approved	CACNA1C
**DB00393**	Nimodipine	Approved	NR3C2
**DB00393**	Nimodipine	Approved	CACNB2
**DB00393**	Nimodipine	Approved	CACNA1S
**DB00393**	Nimodipine	Approved	CACNB4
**DB00393**	Nimodipine	Approved	CACNA1F
**DB00393**	Nimodipine	Approved	CACNB3
**DB00393**	Nimodipine	Approved	CACNA1D
**DB00393**	Nimodipine	Approved	AHR
**DB00421**	Spironolactone	Approved	AR
**DB00421**	Spironolactone	Approved	NR3C2
**DB00436**	Bendroflumethiazide	Approved	CA1
**DB00436**	Bendroflumethiazide	Approved	CA2
**DB00436**	Bendroflumethiazide	Approved	CA4
**DB00436**	Bendroflumethiazide	Approved	KCNMA1
**DB00436**	Bendroflumethiazide	Approved	SLC12A3
**DB00519**	Trandolapril	Approved	ACE
**DB00524**	Metolazone	Approved	SLC12A3
**DB00542**	Benazepril	Approved, Investigational	ACE

**DB00584**	Enalapril	Approved	ACE
**DB00594**	Amiloride	Approved	SCNN1B
**DB00594**	Amiloride	Approved	SCNN1A
**DB00594**	Amiloride	Approved	ABP1
**DB00594**	Amiloride	Approved	ACCN2
**DB00594**	Amiloride	Approved	ACCN1
**DB00594**	Amiloride	Approved	PLAU
**DB00594**	Amiloride	Approved	SLC9A1
**DB00594**	Amiloride	Approved	SCNN1D
**DB00594**	Amiloride	Approved	SCNN1G
**DB00606**	Cyclothiazide	Approved	FXYD2
**DB00606**	Cyclothiazide	Approved	CA1
**DB00606**	Cyclothiazide	Approved	CA2
**DB00606**	Cyclothiazide	Approved	CA4
**DB00622**	Nicardipine	Approved	CHRM1
**DB00622**	Nicardipine	Approved	CHRM3
**DB00622**	Nicardipine	Approved	CHRM5
**DB00622**	Nicardipine	Approved	CHRM4
**DB00622**	Nicardipine	Approved	CACNA2D1
**DB00622**	Nicardipine	Approved	ADRA1D
**DB00622**	Nicardipine	Approved	CALM1
**DB00622**	Nicardipine	Approved	CHRM2
**DB00622**	Nicardipine	Approved	CACNA1C
**DB00622**	Nicardipine	Approved	ADRA1B
**DB00622**	Nicardipine	Approved	ADRA1A
**DB00622**	Nicardipine	Approved	CACNB2
**DB00622**	Nicardipine	Approved	CACNA1D
**DB00622**	Nicardipine	Approved	PDE1A
**DB00622**	Nicardipine	Approved	PDE1B

**DB00641**	Simvastatin	Approved	ITGB2
**DB00641**	Simvastatin	Approved	HMGCR
**DB00653**	Magnesium Sulfate	Approved	CACNB1
**DB00653**	Magnesium Sulfate	Approved	CACNG1
**DB00653**	Magnesium Sulfate	Approved	CACNA2D1
**DB00653**	Magnesium Sulfate	Approved	CACNA1C
**DB00653**	Magnesium Sulfate	Approved	CACNB2
**DB00653**	Magnesium Sulfate	Approved	CACNA1S
**DB00682**	Warfarin	Approved	VKORC1
**DB00691**	Moexipril	Approved	ACE
**DB00691**	Moexipril	Approved	ACE2
**DB00695**	Furosemide	Approved	CA2
**DB00695**	Furosemide	Approved	SLC12A1
**DB00758**	Clopidogrel	Approved, Nutraceutical	P2RY12
**DB00774**	Hydroflumethiazide	Approved	CA1
**DB00774**	Hydroflumethiazide	Approved	CA2
**DB00774**	Hydroflumethiazide	Approved	CA4
**DB00774**	Hydroflumethiazide	Approved	KCNMA1
**DB00774**	Hydroflumethiazide	Approved	ATP1A1
**DB00774**	Hydroflumethiazide	Approved	SLC12A1
**DB00774**	Hydroflumethiazide	Approved	CA12
**DB00774**	Hydroflumethiazide	Approved	CA9
**DB00775**	Tirofiban	Approved	ITGA2B
**DB00775**	Tirofiban	Approved	ITGB3
**DB00790**	Perindopril	Approved	ACE
**DB00796**	Candesartan	Approved	AGTR1
**DB00806**	Pentoxifylline	Approved, Investigational	ADORA1
**DB00806**	Pentoxifylline	Approved, Investigational	PDE5A
**DB00806**	Pentoxifylline	Approved, Investigational	ADORA2A

**DB00806**	Pentoxifylline	Approved, Investigational	PDE4B
**DB00806**	Pentoxifylline	Approved, Investigational	NT5E
**DB00806**	Pentoxifylline	Approved, Investigational	PDE4A
**DB00808**	Indapamide	Approved	KCNQ1
**DB00808**	Indapamide	Approved	KCNE1
**DB00819**	Acetazolamide	Approved	CA1
**DB00819**	Acetazolamide	Approved	CA2
**DB00819**	Acetazolamide	Approved	CA4
**DB00819**	Acetazolamide	Approved	AQP1
**DB00819**	Acetazolamide	Approved	CA3
**DB00819**	Acetazolamide	Approved	CA7
**DB00819**	Acetazolamide	Approved	CA14
**DB00829**	Diazepam	Approved, Illicit	GABRR1
**DB00829**	Diazepam	Approved, Illicit	GABRA2
**DB00829**	Diazepam	Approved, Illicit	GABRA3
**DB00829**	Diazepam	Approved, Illicit	TSPO
**DB00829**	Diazepam	Approved, Illicit	GABRA5
**DB00829**	Diazepam	Approved, Illicit	GABRA1
**DB00829**	Diazepam	Approved, Illicit	GABRB1
**DB00829**	Diazepam	Approved, Illicit	GABRB3
**DB00829**	Diazepam	Approved, Illicit	GABRB2
**DB00829**	Diazepam	Approved, Illicit	GABRG2
**DB00829**	Diazepam	Approved, Illicit	GABRG1
**DB00829**	Diazepam	Approved, Illicit	GABRG3
**DB00829**	Diazepam	Approved, Illicit	GABRE
**DB00829**	Diazepam	Approved, Illicit	GABRP
**DB00829**	Diazepam	Approved, Illicit	GABRQ
**DB00829**	Diazepam	Approved, Illicit	GABRR2
**DB00829**	Diazepam	Approved, Illicit	GABRD

**DB00829**	Diazepam	Approved, Illicit	GABRR3
**DB00876**	Eprosartan	Approved	AGTR1
**DB00880**	Chlorothiazide	Approved	CA1
**DB00880**	Chlorothiazide	Approved	CA2
**DB00880**	Chlorothiazide	Approved	CA4
**DB00880**	Chlorothiazide	Approved	SLC12A3
**DB00881**	Quinapril	Approved, Investigational	ACE
**DB00887**	Bumetanide	Approved	SLC12A5
**DB00887**	Bumetanide	Approved	SLC12A2
**DB00887**	Bumetanide	Approved	SLC12A4
**DB00887**	Bumetanide	Approved	SLC12A1
**DB00887**	Bumetanide	Approved	CFTR
**DB00903**	Ethacrynic acid	Approved	ATP1A1
**DB00903**	Ethacrynic acid	Approved	SLC12A1
**DB00945**	Acetylsalicylic acid	Approved	AKR1C1
**DB00945**	Acetylsalicylic acid	Approved	PTGS2
**DB00945**	Acetylsalicylic acid	Approved	PTGS1
**DB00975**	Dipyridamole	Approved	PDE5A
**DB00975**	Dipyridamole	Approved	PDE10A
**DB00975**	Dipyridamole	Approved	PDE4A
**DB00975**	Dipyridamole	Approved	ADA
**DB00999**	Hydrochlorothiazide	Approved	CA1
**DB00999**	Hydrochlorothiazide	Approved	CA2
**DB00999**	Hydrochlorothiazide	Approved	CA4
**DB00999**	Hydrochlorothiazide	Approved	KCNMA1
**DB00999**	Hydrochlorothiazide	Approved	SLC12A3
**DB00999**	Hydrochlorothiazide	Approved	CA12
**DB00999**	Hydrochlorothiazide	Approved	CA9
**DB01021**	Trichlormethiazide	Approved	CA1

**DB01021**	Trichlormethiazide	Approved	CA2
**DB01021**	Trichlormethiazide	Approved	CA4
**DB01021**	Trichlormethiazide	Approved	ATP1A1
**DB01021**	Trichlormethiazide	Approved	SLC12A1
**DB01029**	Irbesartan	Approved, Investigational	AGTR1
**DB01029**	Irbesartan	Approved, Investigational	JUN
**DB01050**	Ibuprofen	Approved	PTGS1
**DB01050**	Ibuprofen	Approved	PPARG
**DB01050**	Ibuprofen	Approved	BCL2
**DB01050**	Ibuprofen	Approved	PTGS2
**DB01050**	Ibuprofen	Approved	CFTR
**DB01050**	Ibuprofen	Approved	PLAT
**DB01050**	Ibuprofen	Approved	THBD
**DB01050**	Ibuprofen	Approved	FABP2
**DB01054**	Nitrendipine	Approved	CACNG1
**DB01054**	Nitrendipine	Approved	CACNA2D1
**DB01054**	Nitrendipine	Approved	CACNA1H
**DB01054**	Nitrendipine	Approved	CACNA1C
**DB01054**	Nitrendipine	Approved	CACNB2
**DB01054**	Nitrendipine	Approved	CACNA1S
**DB01054**	Nitrendipine	Approved	CACNA1D
**DB01054**	Nitrendipine	Approved	CACNA2D2
**DB01076**	Atorvastatin	Approved	DPP4
**DB01076**	Atorvastatin	Approved	HMGCR
**DB01076**	Atorvastatin	Approved	AHR
**DB01095**	Fluvastatin	Approved	HMGCR
**DB01098**	Rosuvastatin	Approved	HMGCR
**DB01109**	Heparin	Approved, Investigational	F10
**DB01109**	Heparin	Approved, Investigational	SERPINC1

**DB01109**	Heparin	Approved, Investigational	SELP
**DB01166**	Cilostazol	Approved	PDE3A
**DB01183**	Naloxone	Approved	ESR1
**DB01183**	Naloxone	Approved	OPRD1
**DB01183**	Naloxone	Approved	OPRM1
**DB01183**	Naloxone	Approved	CREB1
**DB01183**	Naloxone	Approved	OPRK1
**DB01183**	Naloxone	Approved	TLR4
**DB01197**	Captopril	Approved	MMP9
**DB01197**	Captopril	Approved	ACE
**DB01197**	Captopril	Approved	MMP2
**DB01225**	Enoxaparin	Approved	F10
**DB01225**	Enoxaparin	Approved	SERPINC1
**DB01340**	Cilazapril	Approved	ACE
**DB01599**	Probucol	Approved	ABCA1
**DB01599**	Probucol	Approved	CES1
**DB04841**	Flunarizine	Approved	CACNA1I
**DB04841**	Flunarizine	Approved	CACNA1H
**DB04841**	Flunarizine	Approved	CALM1
**DB04841**	Flunarizine	Approved	HRH1
**DB04841**	Flunarizine	Approved	CACNA1G
**DB06209**	Prasugrel	Approved	P2RY12
**DB06228**	Rivaroxaban	Approved	F10
**DB06605**	Apixaban	Approved	F10
**DB06693**	Mevastatin	Approved	HMGCR
**DB06695**	Dabigatran etexilate	Approved	F2
**DB06779**	Dalteparin	Approved	VEGFA
**DB06779**	Dalteparin	Approved	SERPINC1
**DB06779**	Dalteparin	Approved	TFPI

**DB06779**	Dalteparin	Approved	SELP
**DB06822**	Tinzaparin	Approved	SERPINC1
**DB06822**	Tinzaparin	Approved	ITGA4
**DB06822**	Tinzaparin	Approved	CXCL12
**DB08813**	Nadroparin	Approved	SERPINC1
**DB08813**	Nadroparin	Approved	FOS
**DB08813**	Nadroparin	Approved	SELP
**DB08814**	Triflusal	Approved	NOS2
**DB08814**	Triflusal	Approved	PTGS1
**DB08814**	Triflusal	Approved	PDE10A
**DB08814**	Triflusal	Approved	NFKB1
**DB08816**	Ticagrelor	Approved	P2RY12
**DB08836**	Temocapril	Investigational	ACE

Table S3. GO enrichment analysis-Biological process terms of AIS genes. Here we showed the toppest 200 Biological process terms of AIS credible genes. The PV and CPV respectively mean P-value and Corrected p-value.

GO-ID	Description	PV	CPV
**GO:0006950**	response to stress	2.81E-60	1.13E-56
**GO:0042221**	response to chemical stimulus	6.37E-53	1.28E-49
**GO:0043067**	regulation of programmed cell death	1.03E-47	1.37E-44
**GO:0042981**	regulation of apoptosis	1.93E-47	1.94E-44
**GO:0010941**	regulation of cell death	2.72E-47	2.19E-44
**GO:0051239**	regulation of multicellular organismal process	1.04E-45	6.94E-43
**GO:0065008**	regulation of biological quality	1.74E-45	9.99E-43
**GO:0050896**	response to stimulus	8.44E-45	4.23E-42
**GO:0009611**	response to wounding	1.92E-41	8.55E-39
**GO:0048518**	positive regulation of biological process	1.6E-39	6.44E-37

**GO:0010033**	response to organic substance	4.22E-37	1.54E-34
**GO:0048522**	positive regulation of cellular process	7.05E-35	2.36E-32
**GO:0060548**	negative regulation of cell death	3.97E-34	1.17E-31
**GO:0065007**	biological regulation	4.07E-34	1.17E-31
**GO:0048519**	negative regulation of biological process	6.32E-34	1.69E-31
**GO:0043066**	negative regulation of apoptosis	1.18E-32	2.96E-30
**GO:0043069**	negative regulation of programmed cell death	2.99E-32	7.06E-30
**GO:0042592**	homeostatic process	8.27E-30	1.84E-27
**GO:0023052**	signaling	1.61E-29	3.41E-27
**GO:0048731**	system development	1.89E-29	3.8E-27
**GO:0048856**	anatomical structure development	2.25E-29	4.3E-27
**GO:0032501**	multicellular organismal process	7.41E-29	1.35E-26
**GO:0048523**	negative regulation of cellular process	1.04E-28	1.81E-26
**GO:0032502**	developmental process	9.9E-28	1.66E-25
**GO:0051240**	positive regulation of multicellular organismal process	2.83E-27	4.55E-25
**GO:0044057**	regulation of system process	4.71E-26	7.28E-24
**GO:0050789**	regulation of biological process	6.59E-26	9.79E-24
**GO:0048583**	regulation of response to stimulus	8.61E-26	1.23E-23
**GO:0048878**	chemical homeostasis	1.43E-25	1.99E-23
**GO:0032879**	regulation of localization	1.61E-25	2.15E-23
**GO:0007275**	multicellular organismal development	1.67E-25	2.16E-23
**GO:0010646**	regulation of cell communication	3.69E-25	4.63E-23
**GO:0009605**	response to external stimulus	5.2E-25	6.32E-23
**GO:0006916**	anti-apoptosis	6.21E-25	7.34E-23
**GO:0051241**	negative regulation of multicellular organismal process	6.49E-25	7.44E-23
**GO:0032101**	regulation of response to external stimulus	7.72E-25	8.61E-23
**GO:0042060**	wound healing	5.22E-24	5.66E-22
**GO:0019725**	cellular homeostasis	1.54E-23	1.62E-21
**GO:0065009**	regulation of molecular function	2.01E-23	2.07E-21

**GO:0050794**	regulation of cellular process	4.47E-23	4.49E-21
**GO:0050793**	regulation of developmental process	8.38E-23	8.2E-21
**GO:0009628**	response to abiotic stimulus	3.79E-22	3.62E-20
**GO:0007399**	nervous system development	5.53E-22	5.16E-20
**GO:0023060**	signal transmission	8.03E-22	7.16E-20
**GO:0023046**	signaling process	8.03E-22	7.16E-20
**GO:0051049**	regulation of transport	1.17E-21	1.03E-19
**GO:0043068**	positive regulation of programmed cell death	3.27E-21	2.79E-19
**GO:0010942**	positive regulation of cell death	5.36E-21	4.48E-19
**GO:0044093**	positive regulation of molecular function	7.23E-21	5.92E-19
**GO:0043065**	positive regulation of apoptosis	1.07E-20	8.55E-19
**GO:0023033**	signaling pathway	1.62E-20	1.27E-18
**GO:0007166**	cell surface receptor linked signaling pathway	3.2E-20	2.47E-18
**GO:0007610**	behavior	3.67E-20	2.78E-18
**GO:0051716**	cellular response to stimulus	1.34E-19	9.99E-18
**GO:0050817**	coagulation	1.48E-19	1.06E-17
**GO:0007596**	blood coagulation	1.48E-19	1.06E-17
**GO:0080134**	regulation of response to stress	1.99E-19	1.4E-17
**GO:0050801**	ion homeostasis	2.54E-19	1.76E-17
**GO:0050878**	regulation of body fluid levels	2.6E-19	1.77E-17
**GO:0050804**	regulation of synaptic transmission	2.7E-19	1.81E-17
**GO:0006873**	cellular ion homeostasis	2.85E-19	1.88E-17
**GO:0051094**	positive regulation of developmental process	3.76E-19	2.42E-17
**GO:0050790**	regulation of catalytic activity	3.8E-19	2.42E-17
**GO:0048513**	organ development	5.26E-19	3.27E-17
**GO:0001666**	response to hypoxia	5.38E-19	3.27E-17
**GO:0031644**	regulation of neurological system process	5.38E-19	3.27E-17
**GO:0051050**	positive regulation of transport	6.2E-19	3.68E-17
**GO:0055082**	cellular chemical homeostasis	6.23E-19	3.68E-17

**GO:0007599**	hemostasis	9.39E-19	5.46E-17
**GO:0043085**	positive regulation of catalytic activity	1.54E-18	8.83E-17
**GO:0009607**	response to biotic stimulus	1.6E-18	9.07E-17
**GO:0006979**	response to oxidative stress	2.48E-18	1.38E-16
**GO:0051969**	regulation of transmission of nerve impulse	3.46E-18	1.89E-16
**GO:0030334**	regulation of cell migration	3.48E-18	1.89E-16
**GO:0070482**	response to oxygen levels	4.26E-18	2.28E-16
**GO:0000302**	response to reactive oxygen species	1.01E-17	5.33E-16
**GO:0007154**	cell communication	3.15E-17	1.64E-15
**GO:0040012**	regulation of locomotion	3.92E-17	2.02E-15
**GO:0009991**	response to extracellular stimulus	4.37E-17	2.22E-15
**GO:0043523**	regulation of neuron apoptosis	5.12E-17	2.54E-15
**GO:0042127**	regulation of cell proliferation	5.17E-17	2.54E-15
**GO:0006952**	defense response	5.19E-17	2.54E-15
**GO:0070887**	cellular response to chemical stimulus	5.33E-17	2.58E-15
**GO:0042493**	response to drug	7.18E-17	3.43E-15
**GO:0051789**	response to protein stimulus	8.31E-17	3.92E-15
**GO:0048869**	cellular developmental process	1.23E-16	5.75E-15
**GO:0051270**	regulation of cellular component movement	1.63E-16	7.54E-15
**GO:0051246**	regulation of protein metabolic process	1.9E-16	8.66E-15
**GO:0030154**	cell differentiation	2.01E-16	9.08E-15
**GO:0007268**	synaptic transmission	2.27E-16	1.01E-14
**GO:0045595**	regulation of cell differentiation	3.29E-16	1.45E-14
**GO:0042391**	regulation of membrane potential	3.76E-16	1.64E-14
**GO:0006954**	inflammatory response	3.88E-16	1.67E-14
**GO:0050818**	regulation of coagulation	4.52E-16	1.93E-14
**GO:0019226**	transmission of nerve impulse	4.57E-16	1.93E-14
**GO:0009653**	anatomical structure morphogenesis	5.62E-16	2.35E-14
**GO:0010035**	response to inorganic substance	7.83E-16	3.24E-14

**GO:0009719**	response to endogenous stimulus	8.9E-16	3.65E-14
**GO:0030193**	regulation of blood coagulation	1.04E-15	4.2E-14
**GO:0010647**	positive regulation of cell communication	1.07E-15	4.29E-14
**GO:0031667**	response to nutrient levels	1.66E-15	6.58E-14
**GO:0048514**	blood vessel morphogenesis	1.77E-15	6.95E-14
**GO:0042542**	response to hydrogen peroxide	1.84E-15	7.18E-14
**GO:0032268**	regulation of cellular protein metabolic process	1.87E-15	7.2E-14
**GO:0003013**	circulatory system process	2.84E-15	1.08E-13
**GO:0008015**	blood circulation	2.84E-15	1.08E-13
**GO:0048699**	generation of neurons	3.32E-15	1.24E-13
**GO:0050727**	regulation of inflammatory response	4.18E-15	1.55E-13
**GO:0001568**	blood vessel development	8.26E-15	3.04E-13
**GO:0061041**	regulation of wound healing	8.74E-15	3.19E-13
**GO:0022008**	neurogenesis	9.55E-15	3.45E-13
**GO:0031099**	regeneration	1.73E-14	6.18E-13
**GO:0007267**	cell-cell signaling	1.84E-14	6.54E-13
**GO:0001944**	vasculature development	2.38E-14	8.38E-13
**GO:0008219**	cell death	2.72E-14	9.5E-13
**GO:0042325**	regulation of phosphorylation	3.21E-14	1.11E-12
**GO:0009266**	response to temperature stimulus	3.65E-14	1.25E-12
**GO:0007165**	signal transduction	3.78E-14	1.29E-12
**GO:0016265**	death	3.92E-14	1.32E-12
**GO:0032496**	response to lipopolysaccharide	3.95E-14	1.32E-12
**GO:0002682**	regulation of immune system process	4.7E-14	1.56E-12
**GO:0002237**	response to molecule of bacterial origin	5.73E-14	1.89E-12
**GO:0051338**	regulation of transferase activity	5.95E-14	1.94E-12
**GO:0009725**	response to hormone stimulus	6.96E-14	2.25E-12
**GO:0051174**	regulation of phosphorus metabolic process	7.11E-14	2.26E-12
**GO:0019220**	regulation of phosphate metabolic process	7.11E-14	2.26E-12

**GO:0014070**	response to organic cyclic substance	7.72E-14	2.44E-12
**GO:0051347**	positive regulation of transferase activity	1.96E-13	6.16E-12
**GO:0043549**	regulation of kinase activity	2.03E-13	6.31E-12
**GO:0008217**	regulation of blood pressure	2.48E-13	7.65E-12
**GO:0033674**	positive regulation of kinase activity	2.92E-13	8.95E-12
**GO:0044092**	negative regulation of molecular function	3.21E-13	9.77E-12
**GO:0051098**	regulation of binding	3.65E-13	1.1E-11
**GO:0031325**	positive regulation of cellular metabolic process	3.69E-13	1.11E-11
**GO:0009893**	positive regulation of metabolic process	3.75E-13	1.12E-11
**GO:0045860**	positive regulation of protein kinase activity	4.32E-13	1.27E-11
**GO:0006915**	apoptosis	4.9E-13	1.44E-11
**GO:0045859**	regulation of protein kinase activity	7.6E-13	2.21E-11
**GO:0007611**	learning or memory	9.69E-13	2.8E-11
**GO:0030182**	neuron differentiation	1.02E-12	2.91E-11
**GO:0012501**	programmed cell death	1.06E-12	3E-11
**GO:0035466**	regulation of signaling pathway	1.06E-12	3E-11
**GO:0045429**	positive regulation of nitric oxide biosynthetic process	1.5E-12	4.2E-11
**GO:0002376**	immune system process	2.1E-12	5.85E-11
**GO:0001817**	regulation of cytokine production	2.23E-12	6.17E-11
**GO:0048666**	neuron development	2.3E-12	6.33E-11
**GO:0048545**	response to steroid hormone stimulus	2.38E-12	6.51E-11
**GO:0045428**	regulation of nitric oxide biosynthetic process	2.43E-12	6.6E-11
**GO:0009892**	negative regulation of metabolic process	2.55E-12	6.87E-11
**GO:0048167**	regulation of synaptic plasticity	2.67E-12	7.15E-11
**GO:0009891**	positive regulation of biosynthetic process	2.71E-12	7.22E-11
**GO:0050819**	negative regulation of coagulation	2.89E-12	7.62E-11
**GO:0030335**	positive regulation of cell migration	3.45E-12	9.04E-11
**GO:0090066**	regulation of anatomical structure size	3.61E-12	9.42E-11
**GO:0031328**	positive regulation of cellular biosynthetic process	3.82E-12	9.9E-11

**GO:0051046**	regulation of secretion	3.92E-12	1.01E-10
**GO:0045597**	positive regulation of cell differentiation	4.77E-12	1.22E-10
**GO:0030194**	positive regulation of blood coagulation	6.8E-12	1.73E-10
**GO:0051704**	multi-organism process	8.86E-12	2.24E-10
**GO:0051047**	positive regulation of secretion	1.04E-11	2.6E-10
**GO:0002685**	regulation of leukocyte migration	1.05E-11	2.62E-10
**GO:0022603**	regulation of anatomical structure morphogenesis	1.09E-11	2.71E-10
**GO:0051899**	membrane depolarization	1.15E-11	2.82E-10
**GO:0032103**	positive regulation of response to external stimulus	1.31E-11	3.21E-10
**GO:0051272**	positive regulation of cellular component movement	1.76E-11	4.25E-10
**GO:0040017**	positive regulation of locomotion	1.76E-11	4.25E-10
**GO:0048468**	cell development	1.87E-11	4.49E-10
**GO:0030195**	negative regulation of blood coagulation	2.03E-11	4.86E-10
**GO:0009617**	response to bacterium	2.12E-11	5.03E-10
**GO:0031175**	neuron projection development	2.55E-11	6.02E-10
**GO:0010627**	regulation of intracellular protein kinase cascade	2.63E-11	6.17E-10
**GO:0006800**	oxygen and reactive oxygen species metabolic process	3.2E-11	7.47E-10
**GO:0006986**	response to unfolded protein	4.24E-11	9.85E-10
**GO:0002687**	positive regulation of leukocyte migration	4.78E-11	1.1E-09
**GO:0051093**	negative regulation of developmental process	5.02E-11	1.15E-09
**GO:0012502**	induction of programmed cell death	5.05E-11	1.15E-09
**GO:0009967**	positive regulation of signal transduction	5.14E-11	1.17E-09
**GO:0055066**	di-, tri-valent inorganic cation homeostasis	6.99E-11	1.58E-09
**GO:0055074**	calcium ion homeostasis	7.11E-11	1.59E-09
**GO:0023056**	positive regulation of signaling process	8.72E-11	1.95E-09
**GO:0009966**	regulation of signal transduction	9.06E-11	2.01E-09
**GO:0055065**	metal ion homeostasis	9.21E-11	2.03E-09
**GO:0035468**	positive regulation of signaling pathway	1.04E-10	2.29E-09
**GO:0045765**	regulation of angiogenesis	1.05E-10	2.29E-09

**GO:0031399**	regulation of protein modification process	1.08E-10	2.34E-09
**GO:0050820**	positive regulation of coagulation	1.19E-10	2.56E-09
**GO:0023051**	regulation of signaling process	1.2E-10	2.59E-09
**GO:0051173**	positive regulation of nitrogen compound metabolic process	1.25E-10	2.68E-09
**GO:0055080**	cation homeostasis	1.32E-10	2.8E-09
**GO:0006874**	cellular calcium ion homeostasis	1.36E-10	2.88E-09
**GO:0042312**	regulation of vasodilation	1.43E-10	3.01E-09
**GO:0051707**	response to other organism	1.57E-10	3.28E-09
**GO:0006917**	induction of apoptosis	1.73E-10	3.61E-09
**GO:0003008**	system process	1.87E-10	3.87E-09
**GO:0033554**	cellular response to stress	2.02E-10	4.15E-09
**GO:0040008**	regulation of growth	2.04E-10	4.19E-09
**GO:0060284**	regulation of cell development	2.15E-10	4.38E-09
**GO:0032583**	regulation of gene-specific transcription	2.19E-10	4.44E-09
**GO:0050729**	positive regulation of inflammatory response	2.25E-10	4.54E-09
**GO:0060341**	regulation of cellular localization	2.49E-10	5E-09

Table S4. GO enrichment analysis-Molecular Function terms of AIS genes.

**GO-ID**	**Description**	**PV**	**CPV**
**GO:0005102**	receptor binding	7.81E-24	7.64E-21
**GO:0005515**	protein binding	2.43E-22	1.19E-19
**GO:0046983**	protein dimerization activity	1.64E-20	5.35E-18
**GO:0008066**	glutamate receptor activity	7.21E-14	1.76E-11
**GO:0042802**	identical protein binding	2.40E-12	4.71E-10
**GO:0019899**	enzyme binding	6.38E-12	1.04E-09
**GO:0046982**	protein heterodimerization activity	5.04E-11	7.04E-09
**GO:0005234**	extracellular-glutamate-gated ion channel activity	1.19E-10	1.45E-08

**GO:0042803**	protein homodimerization activity	3.01E-10	3.28E-08
**GO:0005231**	excitatory extracellular ligand-gated ion channel activity	4.57E-10	4.48E-08
**GO:0015267**	channel activity	5.83E-10	5.14E-08
**GO:0022803**	passive transmembrane transporter activity	6.30E-10	5.14E-08
**GO:0004970**	ionotropic glutamate receptor activity	9.02E-10	6.79E-08
**GO:0008201**	heparin binding	2.58E-09	1.81E-07
**GO:0005539**	glycosaminoglycan binding	3.37E-09	2.20E-07
**GO:0022838**	substrate-specific channel activity	6.79E-09	4.10E-07
**GO:0016209**	antioxidant activity	7.13E-09	4.10E-07
**GO:0020037**	heme binding	1.41E-08	7.64E-07
**GO:0022891**	substrate-specific transmembrane transporter activity	2.08E-08	1.00E-06
**GO:0030247**	polysaccharide binding	2.23E-08	1.00E-06
**GO:0001871**	pattern binding	2.23E-08	1.00E-06
**GO:0022892**	substrate-specific transporter activity	2.25E-08	1.00E-06
**GO:0015075**	ion transmembrane transporter activity	2.97E-08	1.26E-06
**GO:0005216**	ion channel activity	3.60E-08	1.47E-06
**GO:0046906**	tetrapyrrole binding	4.25E-08	1.67E-06
**GO:0022857**	transmembrane transporter activity	4.74E-08	1.78E-06
**GO:0008083**	growth factor activity	6.46E-08	2.34E-06
**GO:0043498**	cell surface binding	1.47E-07	5.15E-06
**GO:0005230**	extracellular ligand-gated ion channel activity	1.99E-07	6.57E-06
**GO:0022836**	gated channel activity	2.01E-07	6.57E-06
**GO:0005215**	transporter activity	2.27E-07	7.16E-06
**GO:0008324**	cation transmembrane transporter activity	4.17E-07	1.27E-05
**GO:0015276**	ligand-gated ion channel activity	4.93E-07	1.42E-05
**GO:0022834**	ligand-gated channel activity	4.93E-07	1.42E-05
**GO:0032403**	protein complex binding	5.67E-07	1.59E-05
**GO:0070325**	lipoprotein receptor binding	6.69E-07	1.81E-05

**GO:0005488**	binding	6.86E-07	1.81E-05
**GO:0016667**	oxidoreductase activity, acting on sulfur group of donors	1.12E-06	2.88E-05
**GO:0005179**	hormone activity	1.61E-06	3.97E-05
**GO:0005506**	iron ion binding	1.62E-06	3.97E-05
**GO:0005261**	cation channel activity	3.72E-06	8.87E-05
**GO:0002020**	protease binding	4.54E-06	1.06E-04
**GO:0001664**	G-protein-coupled receptor binding	7.45E-06	1.70E-04
**GO:0004972**	N-methyl-D-aspartate selective glutamate receptor activity	8.21E-06	1.83E-04
**GO:0043028**	caspase regulator activity	1.05E-05	2.28E-04
**GO:0004871**	signal transducer activity	1.11E-05	2.32E-04
**GO:0060089**	molecular transducer activity	1.11E-05	2.32E-04
**GO:0005310**	dicarboxylic acid transmembrane transporter activity	1.28E-05	2.60E-04
**GO:0017153**	sodium:dicarboxylate symporter activity	1.31E-05	2.63E-04
**GO:0004175**	endopeptidase activity	1.50E-05	2.93E-04
**GO:0008289**	lipid binding	1.62E-05	3.12E-04
**GO:0030246**	carbohydrate binding	2.28E-05	4.30E-04
**GO:0015277**	kainate selective glutamate receptor activity	2.39E-05	4.34E-04
**GO:0015368**	calcium:cation antiporter activity	2.39E-05	4.34E-04
**GO:0009055**	electron carrier activity	2.53E-05	4.50E-04
**GO:0030234**	enzyme regulator activity	2.68E-05	4.69E-04
**GO:0004857**	enzyme inhibitor activity	2.90E-05	4.98E-04
**GO:0043027**	caspase inhibitor activity	3.89E-05	6.45E-04
**GO:0050750**	low-density lipoprotein receptor binding	3.89E-05	6.45E-04
**GO:0016491**	oxidoreductase activity	4.06E-05	6.62E-04
**GO:0005126**	cytokine receptor binding	4.53E-05	7.08E-04
**GO:0004517**	nitric-oxide synthase activity	4.70E-05	7.08E-04
**GO:0015038**	glutathione disulfide oxidoreductase activity	4.70E-05	7.08E-04

**GO:0016527**	brain-specific angiogenesis inhibitor activity	4.70E-05	7.08E-04
**GO:0034617**	tetrahydrobiopterin binding	4.70E-05	7.08E-04
**GO:0070011**	peptidase activity, acting on L-amino acid peptides	5.20E-05	7.48E-04
**GO:0035326**	enhancer binding	5.20E-05	7.48E-04
**GO:0003705**	RNA polymerase II transcription factor activity, enhancer binding	5.20E-05	7.48E-04
**GO:0016595**	glutamate binding	5.42E-05	7.64E-04
**GO:0015036**	disulfide oxidoreductase activity	5.46E-05	7.64E-04
**GO:0050660**	FAD binding	5.81E-05	8.02E-04
**GO:0001530**	lipopolysaccharide binding	6.13E-05	8.34E-04
**GO:0019900**	kinase binding	6.35E-05	8.52E-04
**GO:0061134**	peptidase regulator activity	8.13E-05	1.08E-03
**GO:0047485**	protein N-terminus binding	8.49E-05	1.11E-03
**GO:0005509**	calcium ion binding	9.01E-05	1.16E-03
**GO:0004601**	peroxidase activity	9.59E-05	1.20E-03
**GO:0016684**	oxidoreductase activity, acting on peroxide as acceptor	9.59E-05	1.20E-03
**GO:0047498**	calcium-dependent phospholipase A2 activity	1.05E-04	1.31E-03
**GO:0008233**	peptidase activity	1.07E-04	1.31E-03
**GO:0015296**	anion:cation symporter activity	1.19E-04	1.44E-03
**GO:0015035**	protein disulfide oxidoreductase activity	1.35E-04	1.59E-03
**GO:0043499**	eukaryotic cell surface binding	1.35E-04	1.59E-03
**GO:0008238**	exopeptidase activity	1.71E-04	1.99E-03
**GO:0015037**	peptide disulfide oxidoreductase activity	1.83E-04	2.04E-03
**GO:0043125**	ErbB-3 class receptor binding	1.83E-04	2.04E-03
**GO:0005432**	calcium:sodium antiporter activity	1.83E-04	2.04E-03
**GO:0034618**	arginine binding	1.83E-04	2.04E-03
**GO:0016597**	amino acid binding	2.17E-04	2.38E-03
**GO:0015491**	cation:cation antiporter activity	2.61E-04	2.83E-03

**GO:0004866**	endopeptidase inhibitor activity	2.63E-04	2.83E-03
**GO:0004197**	cysteine-type endopeptidase activity	2.81E-04	2.99E-03
**GO:0061135**	endopeptidase regulator activity	2.84E-04	2.99E-03
**GO:0005125**	cytokine activity	2.93E-04	3.05E-03
**GO:0030971**	receptor tyrosine kinase binding	2.98E-04	3.07E-03
**GO:0016502**	nucleotide receptor activity	3.11E-04	3.14E-03
**GO:0001614**	purinergic nucleotide receptor activity	3.11E-04	3.14E-03
**GO:0004869**	cysteine-type endopeptidase inhibitor activity	3.70E-04	3.69E-03
**GO:0005178**	integrin binding	4.10E-04	4.06E-03
**GO:0051400**	BH domain binding	4.45E-04	4.23E-03
**GO:0001609**	adenosine receptor activity, G-protein coupled	4.45E-04	4.23E-03
**GO:0001875**	lipopolysaccharide receptor activity	4.45E-04	4.23E-03
**GO:0051787**	misfolded protein binding	4.45E-04	4.23E-03
**GO:0005543**	phospholipid binding	4.53E-04	4.24E-03
**GO:0005313**	L-glutamate transmembrane transporter activity	4.55E-04	4.24E-03
**GO:0030414**	peptidase inhibitor activity	5.02E-04	4.63E-03
**GO:0015298**	solute:cation antiporter activity	5.07E-04	4.64E-03
**GO:0005057**	receptor signaling protein activity	5.74E-04	5.21E-03
**GO:0005516**	calmodulin binding	5.91E-04	5.31E-03
**GO:0001948**	glycoprotein binding	6.00E-04	5.34E-03
**GO:0019901**	protein kinase binding	6.20E-04	5.47E-03
**GO:0015172**	acidic amino acid transmembrane transporter activity	6.63E-04	5.77E-03
**GO:0005507**	copper ion binding	6.66E-04	5.77E-03
**GO:0070851**	growth factor receptor binding	7.25E-04	6.20E-03
**GO:0043176**	amine binding	7.28E-04	6.20E-03
**GO:0019207**	kinase regulator activity	8.13E-04	6.86E-03
**GO:0070513**	death domain binding	8.66E-04	7.19E-03
**GO:0030151**	molybdenum ion binding	8.66E-04	7.19E-03
**GO:0050662**	coenzyme binding	8.94E-04	7.35E-03

**GO:0031406**	carboxylic acid binding	9.56E-04	7.80E-03
**GO:0008237**	metallopeptidase activity	1.01E-03	8.13E-03
**GO:0004620**	phospholipase activity	1.15E-03	9.26E-03
**GO:0005159**	insulin-like growth factor receptor binding	1.27E-03	9.68E-03
**GO:0004392**	heme oxygenase (decyclizing) activity	1.30E-03	9.68E-03
**GO:0070492**	oligosaccharide binding	1.30E-03	9.68E-03
**GO:0043546**	molybdopterin cofactor binding	1.30E-03	9.68E-03
**GO:0004909**	interleukin-1, Type I, activating receptor activity	1.30E-03	9.68E-03
**GO:0031711**	bradykinin receptor binding	1.30E-03	9.68E-03
**GO:0005314**	high-affinity glutamate transmembrane transporter activity	1.30E-03	9.68E-03
**GO:0051434**	BH3 domain binding	1.30E-03	9.68E-03
**GO:0001641**	group II metabotropic glutamate receptor activity	1.30E-03	9.68E-03
**GO:0004051**	arachidonate 5-lipoxygenase activity	1.30E-03	9.68E-03
**GO:0042277**	peptide binding	1.34E-03	9.89E-03
**GO:0022832**	voltage-gated channel activity	1.41E-03	1.02E-02
**GO:0005244**	voltage-gated ion channel activity	1.41E-03	1.02E-02
**GO:0015179**	L-amino acid transmembrane transporter activity	1.44E-03	1.03E-02
**GO:0004931**	extracellular ATP-gated cation channel activity	1.47E-03	1.03E-02
**GO:0050786**	RAGE receptor binding	1.47E-03	1.03E-02
**GO:0035381**	ATP-gated ion channel activity	1.47E-03	1.03E-02
**GO:0051920**	peroxiredoxin activity	1.47E-03	1.03E-02
**GO:0019838**	growth factor binding	1.56E-03	1.08E-02
**GO:0050661**	NADP or NADPH binding	1.94E-03	1.34E-02
**GO:0005343**	organic acid:sodium symporter activity	2.12E-03	1.45E-02
**GO:0005246**	calcium channel regulator activity	2.17E-03	1.48E-02
**GO:0043121**	neurotrophin binding	2.30E-03	1.55E-02
**GO:0004623**	phospholipase A2 activity	2.53E-03	1.70E-02
**GO:0016860**	intramolecular oxidoreductase activity	2.57E-03	1.71E-02

**GO:0010843**	promoter binding	2.60E-03	1.72E-02
**GO:0022829**	wide pore channel activity	2.76E-03	1.81E-02
**GO:0008144**	drug binding	3.04E-03	1.98E-02
**GO:0004091**	carboxylesterase activity	3.06E-03	1.98E-02
**GO:0004955**	prostaglandin receptor activity	3.35E-03	2.13E-02
**GO:0016668**	oxidoreductase activity, acting on sulfur group of donors, NAD or NADP as acceptor	3.35E-03	2.13E-02
**GO:0017127**	cholesterol transporter activity	3.35E-03	2.13E-02
**GO:0044212**	DNA regulatory region binding	3.41E-03	2.15E-02
**GO:0015485**	cholesterol binding	3.45E-03	2.17E-02
**GO:0008047**	enzyme activator activity	3.78E-03	2.18E-02
**GO:0016298**	lipase activity	3.80E-03	2.18E-02
**GO:0015272**	ATP-activated inward rectifier potassium channel activity	3.82E-03	2.18E-02
**GO:0070653**	high-density lipoprotein receptor binding	3.82E-03	2.18E-02
**GO:0004351**	glutamate decarboxylase activity	3.82E-03	2.18E-02
**GO:0008242**	omega peptidase activity	3.82E-03	2.18E-02
**GO:0004971**	alpha-amino-3-hydroxy-5-methyl-4-isoxazole propionate selective glutamate receptor activity	3.82E-03	2.18E-02
**GO:0033691**	sialic acid binding	3.82E-03	2.18E-02
**GO:0004784**	superoxide dismutase activity	3.82E-03	2.18E-02
**GO:0060230**	lipoprotein lipase activator activity	3.82E-03	2.18E-02
**GO:0031708**	endothelin B receptor binding	3.82E-03	2.18E-02
**GO:0031705**	bombesin receptor binding	3.82E-03	2.18E-02
**GO:0005148**	prolactin receptor binding	3.82E-03	2.18E-02
**GO:0016721**	oxidoreductase activity, acting on superoxide radicals as acceptor	3.82E-03	2.18E-02
**GO:0004063**	aryldialkylphosphatase activity	3.82E-03	2.18E-02

**GO:0016705**	oxidoreductase activity, acting on paired donors, with incorporation or reduction of molecular oxygen	3.84E-03	2.18E-02
**GO:0019904**	protein domain specific binding	3.90E-03	2.21E-02
**GO:0019887**	protein kinase regulator activity	4.14E-03	2.33E-02
**GO:0004709**	MAP kinase kinase kinase activity	4.25E-03	2.36E-02
**GO:0001540**	beta-amyloid binding	4.25E-03	2.36E-02
**GO:0004872**	receptor activity	4.37E-03	2.42E-02
**GO:0004954**	prostanoid receptor activity	4.66E-03	2.54E-02
**GO:0004953**	icosanoid receptor activity	4.66E-03	2.54E-02
**GO:0016594**	glycine binding	4.66E-03	2.54E-02
**GO:0005262**	calcium channel activity	4.73E-03	2.56E-02
**GO:0048037**	cofactor binding	4.95E-03	2.66E-02
**GO:0004702**	receptor signaling protein serine/threonine kinase activity	5.20E-03	2.78E-02
**GO:0004888**	transmembrane receptor activity	5.28E-03	2.81E-02
**GO:0004177**	aminopeptidase activity	5.44E-03	2.86E-02
**GO:0048306**	calcium-dependent protein binding	5.44E-03	2.86E-02
**GO:0015248**	sterol transporter activity	6.24E-03	3.23E-02
**GO:0035035**	histone acetyltransferase binding	6.24E-03	3.23E-02
**GO:0005243**	gap junction channel activity	6.24E-03	3.23E-02
**GO:0033130**	acetylcholine receptor binding	7.46E-03	3.67E-02
**GO:0050544**	arachidonic acid binding	7.46E-03	3.67E-02
**GO:0004157**	dihydropyrimidinase activity	7.46E-03	3.67E-02
**GO:0050321**	tau-protein kinase activity	7.46E-03	3.67E-02
**GO:0004945**	angiotensin type II receptor activity	7.46E-03	3.67E-02
**GO:0004974**	leukotriene receptor activity	7.46E-03	3.67E-02
**GO:0051637**	Gram-positive bacterial cell surface binding	7.46E-03	3.67E-02
**GO:0001595**	angiotensin receptor activity	7.46E-03	3.67E-02
**GO:0004064**	arylesterase activity	7.46E-03	3.67E-02

**GO:0004067**	asparaginase activity	7.46E-03	3.67E-02
**GO:0010181**	FMN binding	8.10E-03	3.94E-02
**GO:0015297**	antiporter activity	8.12E-03	3.94E-02
**GO:0003824**	catalytic activity	8.12E-03	3.94E-02
**GO:0008134**	transcription factor binding	8.50E-03	4.10E-02
**GO:0030545**	receptor regulator activity	8.66E-03	4.15E-02
**GO:0005496**	steroid binding	9.58E-03	4.57E-02
**GO:0015300**	solute:solute antiporter activity	9.85E-03	4.66E-02
**GO:0015370**	solute:sodium symporter activity	9.85E-03	4.66E-02
**GO:0005520**	insulin-like growth factor binding	1.01E-02	4.71E-02
**GO:0016504**	peptidase activator activity	1.01E-02	4.71E-02
**GO:0016564**	transcription repressor activity	1.01E-02	4.71E-02
**GO:0070491**	transcription repressor binding	1.02E-02	4.71E-02
**GO:0004622**	lysophospholipase activity	1.02E-02	4.71E-02
**GO:0005344**	oxygen transporter activity	1.02E-02	4.71E-02
**GO:0004879**	ligand-dependent nuclear receptor activity	1.08E-02	4.94E-02
**GO:0015291**	secondary active transmembrane transporter activity	1.10E-02	5.00E-02

Table S5. GO enrichment analysis-Cellular component terms of AIS genes.

**GO-ID**	**Description**	**PV**	**CPV**
**GO:0005615**	extracellular space	2.94E-26	1.34E-23
**GO:0044421**	extracellular region part	4.47E-25	1.02E-22
**GO:0000267**	cell fraction	5.36E-22	8.15E-20
**GO:0044459**	plasma membrane part	2.44E-20	2.79E-18
**GO:0043005**	neuron projection	7.28E-19	6.64E-17
**GO:0005576**	extracellular region	1.07E-17	8.15E-16
**GO:0005626**	insoluble fraction	2.08E-17	1.36E-15
**GO:0005887**	integral to plasma membrane	9.04E-17	5.11E-15
**GO:0031226**	intrinsic to plasma membrane	1.01E-16	5.11E-15

**GO:0005886**	plasma membrane	2.27E-16	1.03E-14
**GO:0009986**	cell surface	2.88E-16	1.19E-14
**GO:0005624**	membrane fraction	9.97E-16	3.79E-14
**GO:0042995**	cell projection	9.85E-14	3.45E-12
**GO:0030425**	dendrite	2.66E-13	8.65E-12
**GO:0005737**	cytoplasm	4.76E-13	1.45E-11
**GO:0045202**	synapse	1.40E-12	4.00E-11
**GO:0014069**	postsynaptic density	2.37E-12	6.36E-11
**GO:0044444**	cytoplasmic part	3.32E-12	8.41E-11
**GO:0031982**	vesicle	3.91E-11	9.39E-10
**GO:0044297**	cell body	4.75E-11	1.01E-09
**GO:0043025**	neuronal cell body	4.75E-11	1.01E-09
**GO:0044456**	synapse part	4.89E-11	1.01E-09
**GO:0031410**	cytoplasmic vesicle	6.09E-11	1.21E-09
**GO:0044463**	cell projection part	1.04E-10	1.98E-09
**GO:0031988**	membrane-bounded vesicle	1.27E-09	2.31E-08
**GO:0008328**	ionotropic glutamate receptor complex	2.00E-09	3.48E-08
**GO:0030424**	axon	2.06E-09	3.48E-08
**GO:0016023**	cytoplasmic membrane-bounded vesicle	3.61E-09	5.87E-08
**GO:0045121**	membrane raft	6.60E-09	1.04E-07
**GO:0042734**	presynaptic membrane	9.67E-09	1.47E-07
**GO:0044309**	neuron spine	2.38E-08	3.39E-07
**GO:0043197**	dendritic spine	2.38E-08	3.39E-07
**GO:0031975**	envelope	3.58E-08	4.95E-07
**GO:0009897**	external side of plasma membrane	4.39E-08	5.88E-07
**GO:0005625**	soluble fraction	6.03E-08	7.85E-07
**GO:0005829**	cytosol	7.51E-08	9.51E-07
**GO:0030141**	stored secretory granule	4.24E-07	5.23E-06
**GO:0045211**	postsynaptic membrane	7.39E-07	8.87E-06

**GO:0032994**	protein-lipid complex	1.49E-06	1.67E-05
**GO:0034358**	plasma lipoprotein particle	1.49E-06	1.67E-05
**GO:0030426**	growth cone	1.50E-06	1.67E-05
**GO:0031967**	organelle envelope	1.72E-06	1.87E-05
**GO:0030427**	site of polarized growth	1.78E-06	1.89E-05
**GO:0017146**	N-methyl-D-aspartate selective glutamate receptor complex	2.38E-06	2.47E-05
**GO:0034385**	triglyceride-rich lipoprotein particle	2.80E-06	2.78E-05
**GO:0034361**	very-low-density lipoprotein particle	2.80E-06	2.78E-05
**GO:0031983**	vesicle lumen	4.08E-06	3.95E-05
**GO:0005901**	caveola	4.15E-06	3.95E-05
**GO:0043235**	receptor complex	4.53E-06	4.22E-05
**GO:0033267**	axon part	6.34E-06	5.78E-05
**GO:0016020**	membrane	7.18E-06	6.42E-05
**GO:0031091**	platelet alpha granule	7.40E-06	6.49E-05
**GO:0042597**	periplasmic space	1.01E-05	8.55E-05
**GO:0030288**	outer membrane-bounded periplasmic space	1.01E-05	8.55E-05
**GO:0034364**	high-density lipoprotein particle	1.11E-05	9.21E-05
**GO:0019717**	synaptosome	1.46E-05	1.19E-04
**GO:0005739**	mitochondrion	1.55E-05	1.24E-04
**GO:0031012**	extracellular matrix	1.86E-05	1.45E-04
**GO:0031093**	platelet alpha granule lumen	1.87E-05	1.45E-04
**GO:0005740**	mitochondrial envelope	1.94E-05	1.48E-04
**GO:0043195**	terminal button	1.99E-05	1.49E-04
**GO:0060205**	cytoplasmic membrane-bounded vesicle lumen	2.34E-05	1.72E-04
**GO:0030054**	cell junction	2.38E-05	1.72E-04
**GO:0042627**	chylomicron	3.01E-05	2.14E-04
**GO:0030313**	cell envelope	4.75E-05	3.28E-04
**GO:0044462**	external encapsulating structure part	4.75E-05	3.28E-04

**GO:0030312**	external encapsulating structure	7.18E-05	4.88E-04
**GO:0034366**	spherical high-density lipoprotein particle	8.55E-05	5.73E-04
**GO:0034363**	intermediate-density lipoprotein particle	1.56E-04	1.03E-03
**GO:0043679**	axon terminus	2.67E-04	1.72E-03
**GO:0044306**	neuron projection terminus	2.67E-04	1.72E-03
**GO:0043198**	dendritic shaft	2.74E-04	1.74E-03
**GO:0042383**	sarcolemma	3.18E-04	1.99E-03
**GO:0031970**	organelle envelope lumen	3.72E-04	2.29E-03
**GO:0042598**	vesicular fraction	3.99E-04	2.43E-03
**GO:0044433**	cytoplasmic vesicle part	4.21E-04	2.52E-03
**GO:0031966**	mitochondrial membrane	4.88E-04	2.89E-03
**GO:0005578**	proteinaceous extracellular matrix	5.10E-04	2.98E-03
**GO:0048786**	presynaptic active zone	7.40E-04	4.27E-03
**GO:0048471**	perinuclear region of cytoplasm	7.86E-04	4.48E-03
**GO:0005635**	nuclear envelope	9.39E-04	5.29E-03
**GO:0044304**	main axon	1.15E-03	6.36E-03
**GO:0044429**	mitochondrial part	1.16E-03	6.36E-03
**GO:0032281**	alpha-amino-3-hydroxy-5-methyl-4-isoxazolepropionic acid selective glutamate receptor complex	1.17E-03	6.36E-03
**GO:0005788**	endoplasmic reticulum lumen	1.36E-03	7.28E-03
**GO:0005792**	microsome	1.83E-03	9.72E-03
**GO:0019867**	outer membrane	1.97E-03	1.03E-02
**GO:0005921**	gap junction	3.25E-03	1.68E-02
**GO:0032059**	bleb	3.43E-03	1.74E-02
**GO:0008282**	ATP-sensitive potassium channel complex	3.43E-03	1.74E-02
**GO:0005741**	mitochondrial outer membrane	3.66E-03	1.84E-02
**GO:0032279**	asymmetric synapse	4.01E-03	1.99E-02
**GO:0031968**	organelle outer membrane	4.49E-03	2.20E-02

**GO:0045177**	apical part of cell	5.20E-03	2.52E-02
**GO:0043204**	perikaryon	5.67E-03	2.69E-02
**GO:0005758**	mitochondrial intermembrane space	5.67E-03	2.69E-02
**GO:0030666**	endocytic vesicle membrane	6.43E-03	3.02E-02
**GO:0005850**	eukaryotic translation initiation factor 2 complex	6.71E-03	3.12E-02
**GO:0034362**	low-density lipoprotein particle	6.98E-03	3.22E-02
**GO:0031974**	membrane-enclosed lumen	7.93E-03	3.62E-02
**GO:0008021**	synaptic vesicle	8.03E-03	3.63E-02
**GO:0031594**	neuromuscular junction	8.39E-03	3.75E-02
**GO:0019866**	organelle inner membrane	9.45E-03	4.19E-02
**GO:0005783**	endoplasmic reticulum	1.02E-02	4.48E-02
**GO:0033268**	node of Ranvier	1.09E-02	4.66E-02
**GO:0031088**	platelet dense granule membrane	1.09E-02	4.66E-02
**GO:0030877**	beta-catenin destruction complex	1.09E-02	4.66E-02

Table S6. Reactome Pathway enrichment analysis of AIS genes.

**Pathway**	**Number of genes**	**PV**	**CPV**
Formation of Fibrin Clot (Clotting Cascade)	16	2.98E-08	3.25E-06
Common Pathway of Fibrin Clot Formation	11	9.48E-07	5.89E-05
Intrinsic Pathway for Apoptosis	13	3.05E-06	0.00016
Neuronal System	39	3.27E-06	0.000168
Unblocking of NMDA receptor, glutamate binding and activation	9	6.56E-06	0.000294
Hemostasis	59	6.99E-06	0.00031
Platelet activation, signaling and aggregation	31	3.15E-05	0.001092
Apoptotic factor-mediated response	6	3.58E-05	0.001221
Response to elevated platelet cytosolic Ca2+	17	4.04E-05	0.001322

MyD88:Mal cascade initiated on plasma membrane	18	4.06E-05	0.001322
Toll Like Receptor TLR6:TLR2 Cascade	18	4.06E-05	0.001322
Toll Like Receptor 2 (TLR2) Cascade	18	5.84E-05	0.001759
Toll Like Receptor TLR1:TLR2 Cascade	18	5.84E-05	0.001759
Cellular responses to stress	34	5.91E-05	0.001767
Platelet degranulation	16	6.68E-05	0.001955
Detoxification of Reactive Oxygen Species	9	7.35E-05	0.002092
MyD88-independent cascade	18	8.26E-05	0.002294
Toll Like Receptor 3 (TLR3) Cascade	18	8.26E-05	0.002294
TRIF-mediated TLR3/TLR4 signaling	18	8.26E-05	0.002294
Regulation of HSF1-mediated heat shock response	8	0.000103	0.002634
NGF-independant TRKA activation	5	0.000105	0.002669
Activated TLR4 signalling	19	0.00012	0.003003
MyD88 cascade initiated on plasma membrane	16	0.000125	0.003087
Toll Like Receptor 10 (TLR10) Cascade	16	0.000125	0.003087
Toll Like Receptor 5 (TLR5) Cascade	16	0.000125	0.003087
TRAF6 mediated induction of NFkB and MAP kinases upon TLR7/8 or 9 activation	16	0.000141	0.003407
Toll Like Receptor 4 (TLR4) Cascade	20	0.000147	0.003527
Transmission across Chemical Synapses	27	0.00016	0.003743
MyD88 dependent cascade initiated on endosome	16	0.000179	0.004105
Toll Like Receptor 7/8 (TLR7/8) Cascade	16	0.000179	0.004105
Activation of TRKA receptors	5	0.000184	0.004163
Pre-NOTCH Processing in the Endoplasmic Reticulum	5	0.000184	0.004163
Signaling by NOTCH	18	0.000216	0.004696
CREB phosphorylation through the activation of CaMKII	7	0.000243	0.005158
Toll Like Receptor 9 (TLR9) Cascade	16	0.000251	0.005258
Toll-Like Receptors Cascades	21	0.000295	0.006067
Interleukin-1 processing	5	0.0003	0.006168

Tandem pore domain potassium channels	6	0.000304	0.006225
Gastrin-CREB signalling pathway via PKC and MAPK	27	0.000371	0.007226
DEx/H-box helicases activate type I IFN and inflammatory cytokines production	6	0.000423	0.008153
Activation of NMDA receptor upon glutamate binding and postsynaptic events	10	0.000434	0.008321
Arachidonic acid metabolism	11	0.000486	0.009126
Extrinsic Pathway of Fibrin Clot Formation	8	0.000548	0.010008
Regulation of Insulin-like Growth Factor (IGF) transport and uptake by Insulin-like Growth Factor Binding Proteins (IGFBPs)	7	0.000683	0.011933
Sodium/Calcium exchangers	5	0.000691	0.012048
Eicosanoid ligand-binding receptors	6	0.000769	0.012773
Signalling by NGF	31	0.000902	0.014501
Activation of caspases through apoptosome-mediated cleavage	4	0.000955	0.015104
Cytochrome c-mediated apoptotic response	4	0.000955	0.015104
SMAC binds to IAPs	4	0.000955	0.015104
SMAC-mediated apoptotic response	4	0.000955	0.015104
SMAC-mediated dissociation of IAP:caspase complexes	4	0.000955	0.015104
Cellular response to heat stress	9	0.000994	0.015501
CRMPs in Sema3A signaling	6	0.001008	0.015651
Inflammasomes	6	0.0013	0.018792
G alpha (q) signalling events	23	0.001363	0.019502
Signaling by PDGF	22	0.001411	0.020048
NOTCH2 intracellular domain regulates transcription	5	0.001858	0.024376
Programmed Cell Death	20	0.001955	0.025355
NGF signalling via TRKA from the plasma membrane	23	0.001979	0.025476
Ras activation uopn Ca2+ infux through NMDA receptor	6	0.002073	0.026325

Scavenging by Class A Receptors	6	0.002073	0.026325
Nucleotide-binding domain, leucine rich repeat containing receptor (NLR) signaling pathways	10	0.002095	0.026533
Transport of inorganic cations/anions and amino acids/oligopeptides	14	0.002438	0.029629
Advanced glycosylation endproduct receptor signaling	5	0.002456	0.029672
Chylomicron-mediated lipid transport	5	0.002456	0.029672
Dissolution of Fibrin Clot	5	0.002456	0.029672
Notch-HLH transcription pathway	5	0.002456	0.029672
PERK regulates gene expression	7	0.002757	0.032561
RIP-mediated NFkB activation via ZBP1	6	0.003151	0.035608
Attenuation phase	5	0.003182	0.035823
Synthesis of Prostaglandins (PG) and Thromboxanes (TX)	5	0.003182	0.035823
CREB phosphorylation through the activation of Ras	7	0.003261	0.036556
Pre-NOTCH Transcription and Translation	7	0.003261	0.036556
Transport of gamma-carboxylated protein precursors from the endoplasmic reticulum to the Golgi apparatus	4	0.00328	0.036732
Apoptosis	18	0.003323	0.036973
HSF1-dependent transactivation	6	0.003824	0.040854
Intrinsic Pathway of Fibrin Clot Formation	6	0.003824	0.040854
Cell surface interactions at the vascular wall	14	0.004373	0.044892
ATF6-alpha activates chaperone genes	4	0.004531	0.046103
Gamma-carboxylation of protein precursors	4	0.004531	0.046103
Regulation of gene expression by Hypoxia-inducible Factor	4	0.004531	0.046103
Removal of aminoterminal propeptides from gamma-carboxylated proteins	4	0.004531	0.046103
MAP kinase activation in TLR cascade	10	0.004664	0.047314

Table S7. The degrees of AIS existing drug targets. There are totally 161 targets in which 152 are included in the String9.1 PPI network. And more than 45% have degrees larger than 50.

AIS drug targes	Degree	AIS drug targes	Degree
VEGFA	345	CACNB3	45
ESR1	328	CACNA2D1	44
JUN	305	PLAT	44
FOS	296	CACNB2	44
CALM1	271	CACNA1H	43
PTGS2	209	HMGCR	43
PPARG	170	PDE5A	41
NFKB1	170	CACNA1I	40
CHRM2	169	ACE	40
HDAC2	165	LRP2	40
CHRM4	164	DPP4	38
OPRM1	163	F10	30
ITGB3	161	MTR	29
AR	158	MUT	27
AGTR1	150	CA2	26
CHRM3	149	SLC9A1	25
ADORA1	148	THBD	23
OPRD1	147	ADA	21
ADRA2A	146	KRT8	21
OPRK1	146	ACE2	17
ADRA2C	145	SERPINA5	17
ADRA2B	145	KCNMA1	16
P2RY12	144	PAH	16
CHRM1	140	MTHFR	16
CREB1	138	NID1	16
ITGB2	135	GABRG2	15
ADRA1B	132	SLC12A2	14
ADRA1D	130	TFPI	14
CHRM5	128	CES1	14
TLR4	127	GABRB2	12
MMP9	125	CACNA2D2	12
MMP2	123	SERPINB2	11
SERPINE1	112	KCNE1	11
ITGA4	112	GABRB1	11
BCL2	109	CA9	11
ITGA2B	105	FXYD2	10

CXCL12	102	GABRA2	10
F2	96	GABRA3	10
ADRB2	94	GABRA5	10
NT5E	92	GABRA1	10
PLG	91	GABRB3	10
PLAU	89	GABRG3	10
CANX	88	AKR1C1	9
TSPO	83	SCNN1B	9
ADRB1	82	ATP1A1	8
ADRB3	79	ST14	8
CFTR	76	SAA1	8
PDE4A	74	PDXK	7
PLAUR	73	SCNN1A	7
FGA	72	MMAA	7
ADORA2A	71	AQP1	7
NOS2	68	ADRA1A	7
APOE	66	SLC12A3	6
SERPINC1	65	SCNN1G	6
KCNQ1	61	SLC12A1	6
CALR	61	NR3C2	6
PDE1A	61	CA4	5
PDE4B	60	HRH1	5
PDE1B	60	MTRR	5
SELP	59	MMACHC	5
ABCA1	58	ACCN1	4
PDE3A	58	VKORC1	4
CACNA1S	56	FABP2	4
AMBP	55	GABRR2	4
CACNA1C	54	CA3	4
ITGAL	53	GABRR1	3
PDE10A	53	SCNN1D	3
AHR	51	CA7	3
CACNA1G	50	CA1	2
CACNA1D	48	ACCN2	2
CACNA1F	47	CLEC3B	2
PTGS1	45	GABRR3	2
CACNB1	45	SLC12A5	1
CACNG1	45	CA12	1
ANXA2	45	GABRE	1
CACNB4	45	GABRD	1

Table S8. Enriched Reactome pathway of the 10 significant modules. Here we list the highly enriched pathways of 10 classic module. PV and CPV respectively are P-value and Corrected P-value. And the in the time line we annoted the time of the events occur in ischemic cascade. "s", "m", "h", "d", "w", "M" and "y" respectively mean second, minutes, hours, days, weeks, months and years.

Module	Pathway	PV	CPV	Time frame
58	Glycerophospholipid biosynthesis	1.82E-39	7.07E-42	mhdwM
58	Acyl chain remodelling of PC	4.74E-28	6.92E-30	
58	Acyl chain remodelling of PE	1.86E-23	3.71E-25	
58	Synthesis of Leukotrienes (LT) and Eoxins (EX)	3.47E-14	1.18E-15	mh
58	Hydrolysis of LPC	2.70E-08	1.42E-09	mh
64	Activation of NMDA receptor upon glutamate binding and postsynaptic events	1.73E-20	1.36E-18	mh
64	Glutamate Binding, Activation of AMPA Receptors and Synaptic Plasticity	3.54E-20	2.63E-18	smh
64	Trafficking of AMPA receptors	3.54E-20	2.63E-18	hd
64	Unblocking of NMDA receptor, glutamate binding and activation	4.17E-19	2.99E-17	mh
64	Depolarization of the Presynaptic Terminal Triggers the Opening of Calcium Channels	1.22E-15	6.96E-14	mh
94	Toll-Like Receptors Cascades	2.00E-19	6.94E-23	hdw
94	MyD88:Mal cascade initiated on plasma membrane	2.10E-14	5.85E-17	mh

94	Toll Like Receptor TLR6:TLR2 Cascade	2.10E-14	5.85E-17	hdw
94	Toll Like Receptor TLR1:TLR2 Cascade	3.09E-14	1.02E-16	hdw
94	Toll Like Receptor 2 (TLR2) Cascade	3.09E-14	1.02E-16	hdw
94	Interleukin-1 signaling	2.09E-12	9.94E-15	hd
97	Cytokine Signaling in Immune system	7.86E-07	2.80E-08	hd
97	Interleukin receptor SHC signaling	0.000191	1.20E-05	hd
97	Activation of the AP-1 family of transcription factors	0.000381	2.58E-05	sh
97	G beta:gamma signalling through PI3Kgamma	0.001171	8.91E-05	
97	Platelet activation, signaling and aggregation	0.001221	9.36E-05	
103	Nucleotide-binding domain, leucine rich repeat containing receptor (NLR) signaling pathways	2.88E-12	3.41E-14	mh
103	Nucleotide-binding domain, leucine rich repeat containing receptor (NLR) signaling pathways	2.88E-12	3.41E-14	mhw
103	BH3-only proteins associate with and inactivate anti-apoptotic BCL-2 members	6.07E-12	7.48E-14	mhw
103	Activation of BH3-only proteins	1.14E-10	1.92E-12	mhw
103	Apoptotic factor-mediated response	9.13E-09	2.04E-10	mhw
121	Signaling by NOTCH1	1.38E-25	4.95E-28	dwMy

121	Signaling by NOTCH1	1.38E-25	4.95E-28	dwMy
121	Signaling by NOTCH1 HD+PEST Domain Mutants in Cancer	1.38E-25	4.95E-28	
121	Activated NOTCH1 Transmits Signal to the Nucleus	7.51E-25	2.96E-27	dwMy
121	Signaling by NOTCH2	7.51E-23	4.04E-25	dwMy
193	Formation of Fibrin Clot (Clotting Cascade)	1.24E-31	1.80E-34	
193	Gamma-carboxylation, transport, and amino-terminal cleavage of proteins	7.50E-15	1.06E-16	
193	Removal of aminoterminal propeptides from gamma-carboxylated proteins	2.07E-13	3.52E-15	
193	Gamma-carboxylation of protein precursors	2.07E-13	3.52E-15	
193	Dissolution of Fibrin Clot	1.61E-12	2.90E-14	
194	Metabolism of Angiotensinogen to Angiotensins	6.69E-11	3.79E-14	
194	Peptide hormone metabolism	6.64E-08	3.01E-10	mhd
194	Degradation of the extracellular matrix	0.005496	0.000193	hwM
194	Activation of Matrix Metalloproteinases	0.010589	0.000438	hd
194	Extracellular matrix organization	0.032326	0.002071	hdwMy
202	HDL-mediated lipid transport	1.37E-06	4.42E-08	hdwMy
202	Retinoid metabolism and transport	1.79E-06	6.21E-08	hd
202	Binding and Uptake of Ligands by Scavenger Receptors	4.91E-05	2.86E-06	hdwM

202	Formation of Fibrin Clot (Clotting Cascade)	4.91E-05	2.86E-06	
202	Scavenging of heme from plasma	8.10E-05	5.00E-06	
202	GRB2:SOS provides linkage to MAPK signaling for Integrins	0.004614	0.000519	hd
244	Tie2 Signaling	3.08E-11	2.92E-14	hdwMy
244	Cell surface interactions at the vascular wall	2.63E-07	1.66E-09	hd
244	NGF signalling via TRKA from the plasma membrane	3.69E-05	5.13E-07	hdwMy
244	NCAM signaling for neurite out-growth	3.69E-05	5.24E-07	hdwMy
244	IGF1R signaling cascade	7.78E-05	1.50E-06	hdwMy

Table S9. M64 Reactome Pathway analysis. There are 81 enriched pathways. 53 pathways involved in the process of ischemic stroke. These events can appear in the stages in minutes(m), hours(h), days(d), weeks(w), month(M) or years(y). The stage a pathways involved is displayed in the "Timing". And "Unblocking of NMDA receptor, glutamate binding and activation" is the sinificant pathway of M64.[19, 20]

Pathway	PV	CPV	Time frame
Activation of NMDA receptor upon glutamate binding and postsynaptic events	1.73E-20	1.36E-18	mh
Glutamate Binding, Activation of AMPA Receptors and Synaptic Plasticity	3.54E-20	2.63E-18	smh
Trafficking of AMPA receptors	3.54E-20	2.63E-18	hd

Unblocking of NMDA receptor, glutamate binding and activation	4.17E-19	2.99E-17	mh
Depolarization of the Presynaptic Terminal Triggers the Opening of Calcium Channels	1.22E-15	6.96E-14	mh
Post NMDA receptor activation events	1.62E-15	8.97E-14	mh
Ras activation uopn Ca2+ infux through NMDA receptor	4.55E-14	2.27E-12	mh
CREB phosphorylation through the activation of CaMKII	5.85E-13	2.78E-11	mh
CREB phosphorylation through the activation of Ras	1.72E-12	7.69E-11	mh
Integration of energy metabolism	2.59E-11	1.01E-09	m
NCAM1 interactions	8.29E-10	2.84E-08	h
Rap1 signalling	6.49E-09	1.97E-07	hd
NCAM signaling for neurite out-growth	7.11E-08	1.84E-06	dwMy
PKA activation	2.28E-07	5.62E-06	mh
PKA activation in glucagon signalling	3.06E-07	7.43E-06	mh
PKA-mediated phosphorylation of CREB	3.06E-07	7.43E-06	mh
Ca2+ activated K+ channels	5.35E-07	1.20E-05	m
Calmodulin induced events	3.62E-06	6.79E-05	h
CaM pathway	3.62E-06	6.79E-05	h
Ca-dependent events	5.14E-06	9.32E-05	h
cGMP effects	6.48E-06	0.000114	h
Transmembrane transport of small molecules	7.07E-06	0.000124	h
Aquaporin-mediated transport	7.76E-06	0.000132	h

DAG and IP3 signaling	8.34E-06	0.00014	h
Potassium Channels	8.89E-06	0.000148	mh
EGFR interacts with phospholipase C-gamma	1.12E-05	0.000183	h
PLC-gamma1 signalling	1.12E-05	0.000183	h
Axon guidance	1.30E-05	0.000207	dwMy
HSF1-dependent transactivation	1.89E-05	0.000294	h
Nitric oxide stimulates guanylate cyclase	2.27E-05	0.000348	h
Activation of Ca-permeable Kainate Receptor	3.83E-05	0.000554	h
Ionotropic activity of Kainate Receptors	3.83E-05	0.000554	mh
Hormone-sensitive lipase (HSL)-mediated triacylglycerol hydrolysis	3.83E-05	0.000554	d
Trafficking of GluR2-containing AMPA receptors	3.83E-05	0.000554	hd
PLC beta mediated events	4.05E-05	0.000583	mh
G-protein mediated events	4.54E-05	0.000645	hd
Phospholipase C-mediated cascade	6.26E-05	0.000867	mh
PKA-mediated phosphorylation of key metabolic factors	0.000102	0.001355	h
Cellular response to heat stress	0.000191	0.002408	h
CREB phosphorylation through the activation of Adenylate Cyclase	0.000215	0.002687	h
Opioid Signalling	0.000828	0.008484	hd
Inwardly rectifying K+ channels	0.000913	0.00917	mh
Activation of Kainate Receptors upon glutamate binding	0.001017	0.010062	mh

Developmental Biology	0.001263	0.011948	dwMy
Downstream signaling of activated FGFR	0.002011	0.017725	hd
Stimuli-sensing channels	0.002059	0.01809	hd
Lipid digestion, mobilization, and transport	0.002167	0.018795	h
Signaling by FGFR	0.003062	0.025326	hd
NGF signalling via TRKA from the plasma membrane	0.003669	0.029059	hdwMy
Hedgehog 'off' state	0.003683	0.029059	h
NrCAM interactions	0.00524	0.038775	h
Signaling by FGFR in disease	0.005454	0.040059	h
Ion channel transport	0.006766	0.046636	hd

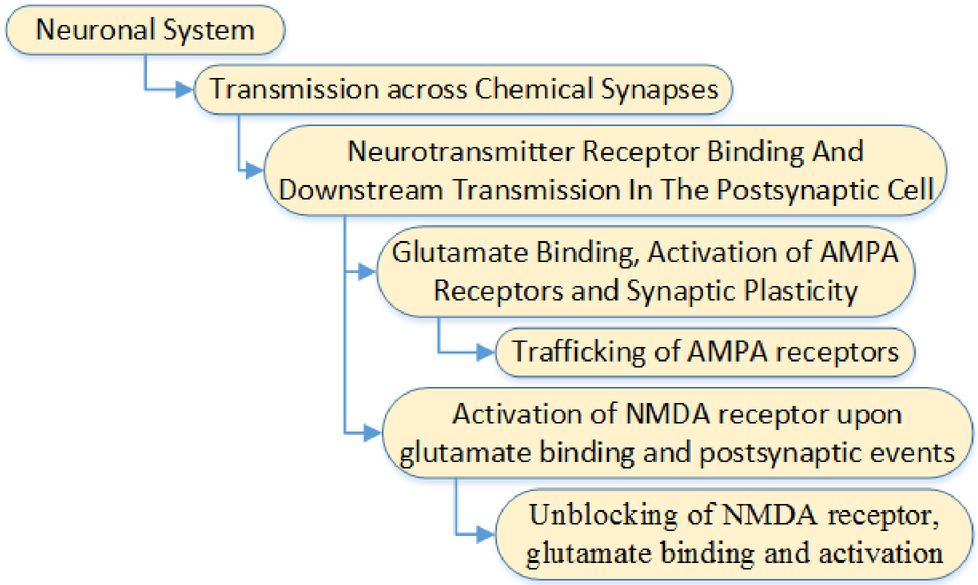

Figure S2. The tree of Reactome pathways of M64. There shows the including and included relationships of enriched Reactome pathways of M64.

Table S10. M145 Reactome Pathway analysis. There are 28 enriched pathways. The 20 pathways in red are shared with the analysis result of credible genes. The most highly enriched pathway "G alpha (i) signalling events" is sinificant pathway of M145.

**Pathway**	**PV**	**CPV**	**Time frame**
G alpha (i) signalling events	2.49E-119	8.99E-116	mh
Class A/1 (Rhodopsin-like receptors)	1.78E-97	3.21E-94	
Defective ACTH causes Obesity and Pro-opiomelanocortinin deficiency (POMCD)	1.94E-84	1.75E-81	
GPCR ligand binding	1.94E-84	1.75E-81	mh
Metabolic disorders of biological oxidation enzymes	2.08E-71	1.50E-68	m
Peptide ligand-binding receptors	1.51E-67	9.10E-65	m
GPCR downstream signaling	1.40E-63	7.23E-61	mh
Signaling by GPCR	1.82E-59	7.32E-57	mh
Signal Transduction	7.81E-33	1.66E-30	
Chemokine receptors bind chemokines	2.81E-32	5.63E-30	h
Amine ligand-binding receptors	3.07E-16	1.73E-14	
Serotonin receptors	2.13E-07	3.82E-06	mh
Nucleotide-like (purinergic) receptors	8.11E-07	1.24E-05	
Lysosphingolipid and LPA receptors	1.56E-06	2.33E-05	
G alpha (z) signalling events	1.71E-05	0.00020565	mh
Activation of C3 and C5	2.54E-05	0.00029046	hd
P2Y receptors	8.89E-05	0.00087765	dwMy
Dopamine receptors	0.00019433	0.00169135	h
Alternative complement activation	0.00019433	0.00169135	dwMy
Formyl peptide receptors bind formyl peptides and many other ligands	0.00040689	0.00312696	
Adrenoceptors	0.00072891	0.00514766	

Regulation of Complement cascade	0.00098933	0.00658094	dwMy
Eicosanoid ligand-binding receptors	0.00252302	0.01446533	
Complement cascade	0.00397903	0.02080628	dwMy
Muscarinic acetylcholine receptors	0.00475556	0.02426144	
Initial triggering of complement	0.00517063	0.02599931	dwMy

Table S11. Novel drugs about neuroprotection of AIS. "Action" means the interaction between drugs and targets. Based on the analysis of M64, There are 21 potential drugs. And 25 potential drugs are screened out based on M145 analysis.

Module	Potential Targets	Action	Novel Drugs
**M64**	CAMK2G	inhibitor	Bosutinib
	GRIA1	antagonist	Perampanel, Enflurane, Isoflurane, Desflurane, Sevoflurane, Methoxyflurane
	GRIA2	antagonist	Hexobarbital, Heptabarbital, Methylphenobarbital, Quinidine barbiturate, Primidone, Pentobarbital, Phenobarbital, Thiopental, Secobarbital
	GRIN1	antagonist	Orphenadrine, Pethidine
	GRIN2A	antagonist	Halothane, Memantine, Felbamate, Pethidine
**M145**	ADORA3	agonist	Adenosine
	DRD2	agonist	Amantadine, Apomorphine, Bromocriptine, Cabergoline, Dopamine, Ergotamine, Ketamine, Levodopa, Lisuride, Minaprine, Pramipexole, Ropinirole, Rotigotine
	HTR1A	agonist	Apomorphine, Bromocriptine, Cabergoline, Cinitapride, Eletriptan, Lisuride, Methysergide, Naratriptan, Ropinirole, Rotigotine, Sumatriptan, Vilazodone, Zolmitriptan
	HTR1D	agonist	Almotriptan, Apomorphine, Bromocriptine, Cabergoline, Dihydroergotamine, Eletriptan, Ergotamine, Frovatriptan,

			Lisuride, Naratriptan, Rizatriptan, Ropinirole, Sumatriptan, Zolmitriptan
	HTR1E	agonist	Eletriptan
	HTR1F	agonist	Eletriptan, Naratriptan, Rizatriptan, Sumatriptan, Zolmitriptan

In addition, there are additional supplementary data files as following, all of which will be supplied if necessary.

Data S1. Identified disease-gene relationships of AIS from Coremine

Data S2. The nodes in 29 modules (OR>2).

Data S3. GO analysis of AIS confirmed genes

Data S4.GO analysis of AIS existing drug targets.

Data S5. GO analysis of 29(OR>2) AIS related modules.

Data S6. Pathway analysis of AIS drug targets.

Data S7. Pathway analysis of 29 AIS related modules.

Reference

1. Jenssen, T.K., et al., A literature network of human genes for high-throughput analysis of gene expression. *Nat Genet* **28** 21-28 (2001).
2. Amberger, J.S., et al., OMIM.org: Online Mendelian Inheritance in Man (OMIM(R)), an online catalog of human genes and genetic disorders. *Nucleic Acids Res* **43** D789-798 (2015).
3. Liu, C.C., et al., DiseaseConnect: a comprehensive web server for mechanism-based disease-disease connections. *Nucleic Acids Res* **42** W137-146 (2014).
4. Franceschini, A., et al., STRING v9.1: protein-protein interaction networks, with increased coverage and integration. *Nucleic Acids Res* **41** D808-815 (2013).
5. Andrus, B. and D. Lacaille, 2013 ACC/AHA guideline on the assessment of cardiovascular risk. *J Am Coll Cardiol* **63** 2886 (2013).
6. Jauch, E.C., et al., Guidelines for the early management of patients with acute ischemic stroke: a guideline for healthcare professionals from the American Heart Association/American Stroke Association. *Stroke* **44** 870-947 (2013).

7. Kernan, W.N., et al., Guidelines for the prevention of stroke in patients with stroke and transient ischemic attack: a guideline for healthcare professionals from the American Heart Association/American Stroke Association. *Stroke* **45** 2160-2236 (2014).
8. Law, V., et al., DrugBank 4.0: shedding new light on drug metabolism. *Nucleic Acids Res* **42** D1091-1097 (2014).
9. Dittrich, M.T., et al., Identifying functional modules in protein-protein interaction networks: an integrated exact approach. *Bioinformatics* **24** i223-231 (2008).
10. Blondel V D, G.J.L., Lambiotte R, et al., Fast unfolding of communities in large networks. *Journal of Statistical Mechanics: Theory and Experiment* **10** P10008 (2008).
11. Ashburner, M., et al., Gene ontology: tool for the unification of biology. The Gene Ontology Consortium. *Nat Genet* **25** 25-29 (2000).
12. Wang, J., et al., NOA: a novel Network Ontology Analysis method. *Nucleic Acids Res* **39** e87 (2011).
13. Maere, S., K. Heymans, and M. Kuiper, BiNGO: a Cytoscape plugin to assess overrepresentation of gene ontology categories in biological networks. *Bioinformatics* **21** 3448-3449 (2005).
14. Smoot, M.E., et al., Cytoscape 2.8: new features for data integration and network visualization. *Bioinformatics* **27** 431-432 (2011).
15. Xie, C., et al., KOBAS 2.0: a web server for annotation and identification of enriched pathways and diseases. *Nucleic Acids Res* **39** W316-322 (2011).
16. Zhou, X., et al., Human symptoms-disease network. *Nat Commun* **5** 4212 (2014).
17. Cormen, T.H., Introduction to algorithms. *MIT press* (2001).
18. Christina G. Towers, A.L.G., Doug S. Micalizzi,w, J. Chuck Harrell,w, Austin E. Gillen,, C.-A.W. Jihye Kim, w, Michael U.J. Oliphant, David J. Drasin,w, Michelle A. Guney, Peter Kabos,, and A.-C.T. Carol A. Sartorius, Charles M. Perou, Joaquin M. Espinosa, Heide L. Ford, The Six1 oncoprotein downregulates p53 via concomitant regulation of RPL26 and microRNA-27a-3p. *Nat Commun* **10.1038** (2015).
19. Barone, F.C. and G.Z. Feuerstein, Inflammatory mediators and stroke: new opportunities for novel therapeutics. *J Cereb Blood Flow Metab* **19** 819-834 (1999).
20. Saenger, A.K. and R.H. Christenson, Stroke biomarkers: progress and challenges for diagnosis, prognosis, differentiation, and treatment. *Clin Chem* **56** 21-33 (2010).
